# Histone acetylation promotes FKBP5 to increase reparative dentin formation in pulp repair

**DOI:** 10.1093/stcltm/szag047

**Published:** 2026-07-28

**Authors:** Shaoying Duan, Qianqian Su, Hui Yang, Xinlei Hu, Aopeng Zhang, Tao Hu, Dan Xie, Ran Cheng

**Affiliations:** State Key Laboratory of Oral Diseases, National Center for Stomatology, National Clinical Research Center for Oral Diseases, Frontier Innovation Center for Dental Medicine Plus, West China Hospital of Stomatology, Sichuan University, Chengdu, 610041, China; State Key Laboratory of Oral Diseases, National Center for Stomatology, National Clinical Research Center for Oral Diseases, Frontier Innovation Center for Dental Medicine Plus, West China Hospital of Stomatology, Sichuan University, Chengdu, 610041, China; State Key Laboratory of Oral Diseases, National Center for Stomatology, National Clinical Research Center for Oral Diseases, Frontier Innovation Center for Dental Medicine Plus, West China Hospital of Stomatology, Sichuan University, Chengdu, 610041, China; Frontier Science Center for Disease Molecular Network, State Key Laboratory of Biotherapy, West China Hospital, Sichuan University, Chengdu, 610041, China; State Key Laboratory of Oral Diseases, National Center for Stomatology, National Clinical Research Center for Oral Diseases, Frontier Innovation Center for Dental Medicine Plus, West China Hospital of Stomatology, Sichuan University, Chengdu, 610041, China; State Key Laboratory of Oral Diseases, National Center for Stomatology, National Clinical Research Center for Oral Diseases, Frontier Innovation Center for Dental Medicine Plus, West China Hospital of Stomatology, Sichuan University, Chengdu, 610041, China; Frontier Science Center for Disease Molecular Network, State Key Laboratory of Biotherapy, West China Hospital, Sichuan University, Chengdu, 610041, China; State Key Laboratory of Oral Diseases, National Center for Stomatology, National Clinical Research Center for Oral Diseases, Frontier Innovation Center for Dental Medicine Plus, West China Hospital of Stomatology, Sichuan University, Chengdu, 610041, China

**Keywords:** H3K9ac, H3K27ac, FKBP5, SAHA, dental pulp stem cells

## Abstract

**Importance:**

A well-formed dentin bridge isolates irritants and prevents bacterial penetration into the remaining pulp tissue, which is essential for pulp regeneration. The existing literature on promoting reparative dentin formation mostly focuses on the effects of pulp-capping materials but lacks investigations into the underlying mechanisms.

**Objective:**

To identify potential targets for enhancing reparative dentin formation as well as the epigenetic mechanisms underlying these effects.

**Design:**

Laboratory-based study combining multi-omics sequencing, epigenetic assays, in vitro FK506-binding protein 51 (FKBP5) knockdown or overexpression experiments, and in vivo validation using rat pulp injury models and nude mouse subcutaneous transplantation models.

**Setting:**

Laboratory-based in vitro and in vivo study.

**Participants:**

N/A

**Intervention(s) or Exposure(s):**

Dental pulp stem cells (DPSCs) were exposed to odontogenic differentiation induction or Lipopolysaccharide (LPS) stimulation. FKBP5 knockdown and overexpression were conducted using lentiviral vectors. In vivo validation was performed using a rat dental pulp injury model and a nude mouse subcutaneous transplantation model combining FKBP5 knockdown or overexpressed DPSCs with treated dentin matrix. Histone acetylation was modulated using dCas9-p300 targeting or pharmacological treatment with suberoylanilide hydroxamic acid (SAHA) or C646.

**Main Outcome(s) and Measure(s):**

FKBP5 expression, mineralized nodule formation, alkaline phosphatase (ALP) activity, odontogenic marker expression, inflammatory cytokine expression, FKBP5 promoter chromatin accessibility, promoter methylation, H3K9ac/H3K27ac enrichment, reparative dentin formation and collagen formation were assessed.

**Results:**

FKBP5 was upregulated during odontogenic differentiation induction and highly expressed in human pulpitis tissues and injured rat pulps. Overexpression of FKBP5 promoting reparative dentin formation, collagen synthesis and mineralization (dentin sialophosphoprotein and dentin matrix protein 1 expressions). Down-regulation of inflammatory factors Interleukin-1β and Interleukin-8 also contributed to the process of pulp repair. H3K9ac and H3K27ac, rather than DNA methylation, were enriched at the FKBP5 promoter, enhancing chromatin accessibility and FKBP5 transcription. SAHA enhanced H3K9ac and H3K27ac and the expression of FKBP5 near the injury site. The newly-formed reparative dentin was upregulated and pulp inflammation was suppressed with the application of SAHA.

**Conclusions and Relevance:**

FKBP5 facilitated reparative dentin and neoplastic collagen synthesis at the injury site while mitigating inflammation. Histone acetylation H3K9 and H3K27 were the mechanism that modulated differentiation effects of FKBP5.

## Graphical Abstract

**Graphical Abstract:**
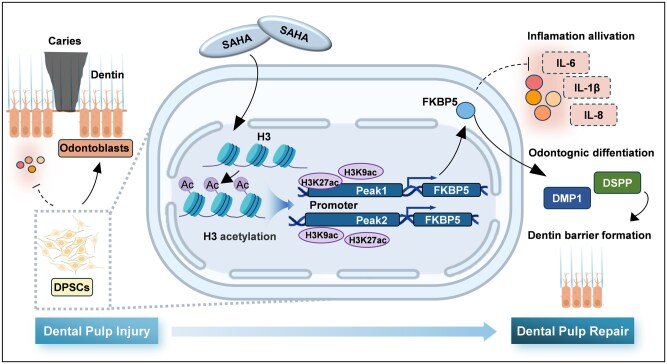

Significance statement A well-formed dentin bridge isolates irritants and prevents bacterial penetration into the remaining pulp tissue, which is essential for pulp regeneration. Our study showed that histone acetylation of H3K9 and H3K27 promoted the expression of FKBP5, thus enhancing reparative dentin and collagen synthesis at the pulp injury site. Our results suggested that enhancing FKBP5 through enhanced histone acetylation was a promising option to facilitate reparative dentin formation.

## Introduction

Pulpitis is an inflammatory disease caused by microbial infection. The associated pain decreases quality of life and imposes both psychological and social burdens.^1^^,^^2^ If untreated, pulpal inflammation can progress to periapical tissues, resulting in periapical inflammation.^3^ Currently, root canal therapy is the clinically preferred treatment to mechanically and chemically debride the infected root canal system, eradicate inflammation, and subsequently fill the root canal. Nevertheless, root canal therapy has limitations. Its success rate is contingent on various factors, with a 25-year cohort study indicating a success rate of ∼85% after 5 years and 60% after 20 years.^4^ After treatment, pulp vitality loss and increased tooth brittleness can cause root fractures and potential tooth loss, with retention rates of ∼90% at 5 years and 50% at 20 years.^4^

Since the 1960s,^5^ preservation, repair, and regeneration of the vital pulp, along with functional restoration, have progressively become a prevailing tendency. In recent years, some researchers have suggested that vital pulp therapy (VPT) may be an alternative to root canal therapy.^6^ A study indicated that the overall efficacy of VPT for irreversible pulpitis in adult permanent teeth was 88% after 12 months.^7^ A comparative analysis of VPT and root canal therapy demonstrated similar success rates.^8^ A dentin bridge is a newly developed hard tissue barrier on the surface of the exposed pulp. When well-established, it secludes irritants and inhibits bacterial infiltration into the residual pulp tissue, which is crucial for pulp regeneration.^9^^,^^10^ However, the rate of dentin bridge formation following pulpotomy in adult permanent teeth is limited. A study revealed that at 12.5 months after pulpotomy in adult permanent molars with irreversible pulpitis, only 37.5% of patients demonstrated dentin bridge formation.^11^ Similarly, 6 weeks after direct pulp capping of adult permanent teeth with mineral trioxide aggregate (MTA) and Biodentine, the rates of calcified bridge formation were 57.7% and 46.1%, respectively.^12^ After 8 weeks of direct pulp capping with TheraCal and MTA, the formation rates of complete dentin bridges in adult third permanent molars were 11% and 56%, respectively.^13^

Pulp regeneration is a multifactorial process. Dental pulp exposure to external stimuli triggers inflammatory responses mediated by pro-inflammatory factors and chemokines, such as interferon gamma, tumor necrosis factor alpha, interleukin 1β (IL-1β), C-X-C Motif Chemokine Ligand 1 (CXCL1), CXCL2, and CXCL8. Conversely, transforming growth factor-β (TGF-β) and IL-10 inhibit these responses and mitigate excessive tissue damage.^14^ Mild levels of pro-inflammatory factors may facilitate differentiation and mineralization, thus improving pulp healing, whereas high levels of pro-inflammatory factors could recruit extra immune cells, exacerbating the inflammatory response.^14–16^ Simultaneously, growth factors like TGF-β and insulin-like growth factors 1 and 2 facilitate cell proliferation and differentiation.^17^ Other essential elements, including dentin salivary protein, dentin phosphoprotein, dentin matrix protein-1 (DMP-1), and osteopontin, can facilitate mineralization.^18^ The vascular endothelial growth factor, basic fibroblast growth factor, and TGF-β promote angiogenesis, reduce inflammatory responses, and facilitate regenerative processes. Most research on pulp regeneration has focused on the mechanisms of pulp regeneration,^19^ with reparative dentin formation primarily investigated in clinical studies utilizing pulp-capping materials, with insufficient research on its underlying mechanism.

Epigenetic modifications, a range of chemical alterations in chromatin DNA and associated proteins that do not modify the DNA sequence, including DNA methylation, histone modification, and non-coding RNAs, are increasingly considered therapeutic targets for various diseases.^20^ For instance, certain HDAC inhibitors, including vorinostat, romidepsin, pabilostat, and belistat, have been approved for the treatment of hematologic diseases.^21^ In addition, epigenetic therapies exhibit considerable potential for pulp regeneration. Our previous study indicated that pan-enhanced histone acetylation regulates neuregulin-1 (NRG1) and enhances regenerative repair of both soft and hard tissues.^22^ Thus, the possible role of epigenetic alterations in the formation of reparative dentin requires further investigation.

We hypothesized that managing reparative dentin formation can shield the residual pulp from stimuli and promote its regeneration. The mobilization of significant targets in the regulation of reparative dentin formation by epigenetic alterations is a potential therapeutic choice. Thus, this work aimed to identify potential targets for enhancing reparative dentin formation as well as the epigenetic mechanisms underlying these effects.

## Methods

### RNA-seq library preparation and sequencing

Dental pulp stem cells (DPSCs) were harvested at 1, 3, 7, and 14 days after odontogenic differentiation (OD) induction and Lipopolysaccharide (LPS) stimulation. Sequencing libraries were generated using the RNA Library Prep Kit Fast RNA-seq Lib Prep Kit V2 (Cat. No. RK20306, ABclonal, China) following the manufacturer’s instructions, and index codes were added to attribute sequences to each sample. First-strand cDNA was synthesized using a random hexamer primer and M-MuLV Reverse Transcriptase. Second-strand cDNA synthesis was subsequently performed using DNA Polymerase I and RNase H. To select cDNA fragments of 370–420 bp in length, library fragments were purified with the AMPure XP system. Then, 3 µL of USER Enzyme was used with size-selected, adaptor-ligated cDNA at 37 °C for 15 min, followed by 5 min at 95 °C. Then, PCR was performed using Phusion High-Fidelity DNA polymerase, Universal PCR primers, and Index Primer. Finally, PCR products were purified, and library quality was assessed on the Agilent 5400 system and quantified using QPCR (1.5 nM). Qualified libraries were pooled and sequenced on Illumina platforms with the PE150 strategy in Novogene Bioinformatics Technology Co., Ltd. (Beijing, China), according to the effective library concentration and data amount required.

### Assay for transposase-accessible chromatin sequencing (ATAC-seq) library construction and sequencing

DPSCs were harvested at 1,3,7, and 14 days after OD induction and LPS stimulation. Nucleus was extracted from the samples, and the nucleus pellet was resuspended in the Tn5 transposase reaction mix. The transposition reaction was incubated at 37 °C for 30 min. After transposition, equimolar amounts of Adapter 1 and Adapter 2 were added, followed by PCR amplification of the library using the Novogene ATAC-seq library preparation kit. After the PCR reaction, libraries were purified using AMPure beads, and library quality was assessed using Qubit. The clustering of the index-coded samples was performed on a cBot Cluster Generation System using TruSeq PE Cluster Kit v3-cBot-HS (Illumina) according to the manufacturer’s instructions. The library preparations were sequenced on the Illumina Novaseq platform at Tianjin Novogene Bioinformatic Technology Co., Ltd. (Beijing, China), and 150 bp paired-end reads were generated.

### Collection of rat tooth germ development samples

Pregnant Sprague-Dawley (SD) rats and neonatal pups were euthanized by isoflurane overdose. Embryonic heads were harvested on gestational days 14 (bud stage) and 16 (cap stage), whereas neonatal heads were collected on postnatal days 1 (bell stage). All specimens were rinsed with saline and fixed in 4% paraformaldehyde for further analysis.

### Establishment of a rat dental pulp injury model

Male SD rats (7 weeks old, ∼220 g) were obtained from Beijing Huafukang Biotechnology Co., Ltd. and anesthetized via isoflurane inhalation. Dental pulp exposure was performed at the mesial fossa of the occlusal surface of the maxillary first molar, which deepened until the pink coloration of the pulp became visible through the dentin at the base of the cavity. Then, the pulp chamber was further enlarged using hand-held K files (#10–#40). Subsequently, 2 μL of LPS (10 mg/mL, L2630, Sigma-Aldrich, USA) from *Escherichia coli* O111: B4 was slowly injected into the pulp cavity using a microinjector. The cavity was then sealed with mixed glass ionomer cement. Rats in the control group underwent no intervention. Maxillary bone samples were collected from control animals and experimental rats at 3 h, 12 h, 1 day, 3 days, and 7 days post-treatment.

### *In vitro* DPSC culture with FKBP5 knockdown or overexpression

Lentiviral vectors for FKBP5 knockdown (sh-FKBP5, GV493) and overexpression (oe-FKBP5, GV492), along with their respective negative controls (sh-NC and oe-NC), were constructed and packaged by Shanghai Genechem. The most effective shRNA sequence (CGAAGGAGCAACAGTAGAAAT) was selected by a preliminary screening. DPSCs were transduced at MOI = 10, and stable cell lines were selected with 2 μg/mL puromycin. Transduction efficiency was evaluated using GFP fluorescence. FKBP5 expression at both mRNA and protein levels was confirmed by qRT-PCR and Western blot, respectively. DPSCs with stable FKBP5 knockdown or overexpression were seeded in 6-well plates at a density of 1 × 10^6^ cells per well and stimulated with 1 µg/mL LPS in culture medium. Treatment groups included LPS + sh-NC, LPS + sh-FKBP5, LPS + oe-NC, and LPS + oe-FKBP5. Inflammatory responses were assessed through subsequent assays. For OD induction, DPSCs were cultured in OD medium for the indicated time points. The corresponding experimental groups included: OD + sh-NC, OD + sh-FKBP5, OD + oe-NC, and OD + oe-FKBP5. The odontogenic potential of DPSCs was evaluated in subsequent analyses.

### Subcutaneous transplantation in nude mice

Four-week-old male BALB/c-nu athymic nude mice (∼15 g; Beijing Huafukang Biotechnology Co., Ltd., China) were anesthetized with isoflurane inhalation under sterile conditions. DPSCs with FKBP5 knockdown or overexpression were cultured for 3 days under OD induction, suspended in Matrigel (1.0 × 10^6^ cells/10 μL; 354248, Corning, USA) and seeded onto human-treated dentin matrix(TDM) (Supplementary Materials). To assess pulp regeneration after 6 weeks, constructs were subcutaneously implanted into the dorsal region of mice. To establish an inflammation-related pulp injury model, FKBP5 knockdown or overexpression was pretreated with 1 µg/mL LPS for 24 h, then combined with Matrigel and transplanted subcutaneously.

### Statistical analysis

All data were analyzed and visualized using GraphPad Prism 8.0 (GraphPad Software, USA). Results are presented as mean ± standard deviation. One-way analysis of variance was applied for comparisons among multiple groups, and an unpaired two-tailed Student’s *t*-test was used for comparisons between two groups. A *P*-value < .05 was considered statistically significant.

## Results

### FKBP5 is a crucial target in OD induction and pulp repair

**Figure 1:**
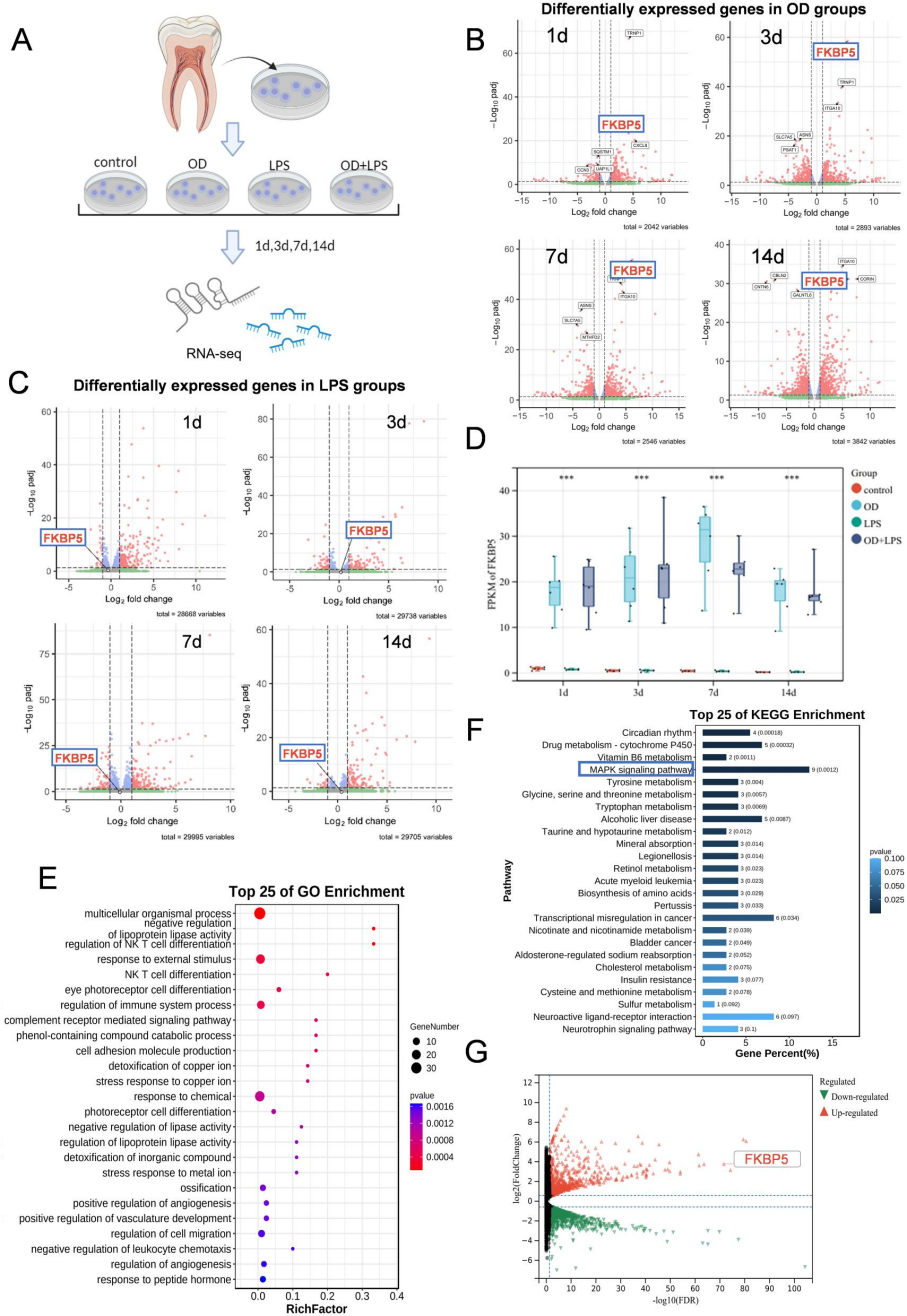
RNA-seq analysis for crucial target identification. (A) Screening flowchart utilizing RNA sequencing analysis following 1, 3, 7, and 14 days of OD induction and LPS stimulation in dental pulp stem cells; (B) Volcano plots of DEGs in DPSCs following OD induction (*N *= 6), featuring annotations for FKBP5, all ranked in the top three; (C) Volcano plots of DEGs in DPSCs after LPS stimulation (*N *= 6), with no differential expression of FKBP5; (D) The comparative expressions of FKBP5 in the OD, LPS, and OD+LPS groups (*N *= 6); (E) Intersection of DEGs of DPSCs at four time points, followed by GO functional enrichment analysis of the top 25 biological processes; (F) KEGG pathway enrichment analysis; (G) Volcano plots showed mRNA expression of oral-derived stem cells after 9 days of OD induction in the GSE244057 dataset from the GEO public database.

Figure 1A illustrates the screening workflow. The top 3 differentially expressed genes (DEGs) at 1, 3, 7, and 14 days of OD induction were *TRNP1*, *FKBP5*, and *CXCL8*; *FKBP5*, *TRNP1*, *IGTA10*; *FKBP5*, *TRNP1*, *IGTA10*; *IGTA10*, *CORIN*, and *FKBP5*, respectively (Figure 1B). FKBP5 was the only protein consistently increased throughout the evaluation period. Nevertheless, at 1, 3, 7, and 14 days after LPS stimulation, no significant alterations in FKBP5 levels were observed compared to the control group (Figure 1C and D). Subsequently, we analyzed DEGs of the OD group throughout the four time periods, identifying 139 identical DEGs. The Gene Ontology (GO) enrichment analysis and Kyoto Encyclopedia of Genes and Genomes (KEGG) pathway enrichment analysis revealed that the DEGs predominantly clustered in the multicellular organism process, response to external stimuli, and response to chemicals categories. The relevant pathways predominantly highlighted osteogenic and dental signaling networks, including MAPK signaling pathways (Figure 1E and F). The RNA-seq dataset GSE244057 from the Gene Expression Omnibus (GEO) database indicated that FKBP5 was highly upregulated 9 days after OD-induction in DPSCs and was a crucial DEG (Figure 1G).

**Figure 2:**
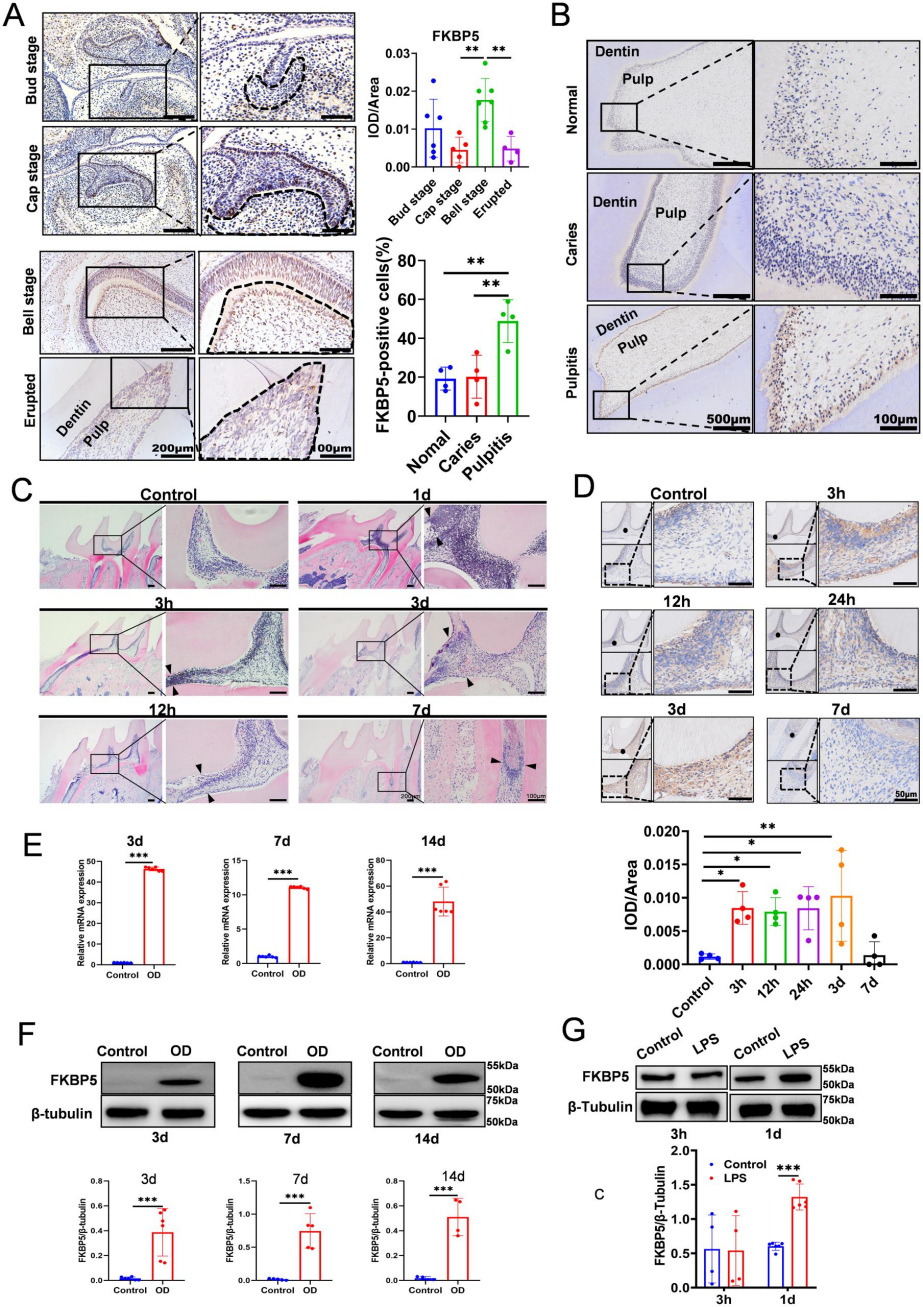
Validation of FKBP5 in rat tooth germ, rat pulp tissues, human pulp tissues, and human DPSCs. (A) IHC staining of FKBP5 in rat tooth germ (*N *= 4–7, **P < .01); (B) IHC staining of FKBP5 in human healthy, carious, and pulpitis pulp tissues (*N *= 4, ns, *P *> .05; *P < .05; **P < .01); (C) HE staining of rat pulp at 3 h, 12 h, 24 h, 3 days, and 7 days after pulp injury; (D) IHC staining of FKBP5 (*N *= 4, *P < .05, **P < .01); (E) the mRNA expression of FKBP5 in DPSCs with OD induction for 3d, 7d, and 14d (*N *= 6, ***P < .001); (F) FKBP5 protein expression in DPSCs with OD induction for 3d, 7d, and 14d (*N *= 4–6, ***P < .001); (G) FKBP5 protein expression in DPSCs with LPS stimulation for 3 h and 1d (*N *= 4–6, ***P < .001).

We further validated FKBP5 expression *in vivo* and *in vitro*. FKBP5 showed weak positive expression in the mesenchymal cell layer during the bud and cap stages of rat odontogenesis (Figure 2A). FKBP5 expression increased throughout the campanulate phase, with significant FKBP5 localization around the dental papilla. FKBP5 expression was negligible in the pulp tissue of the erupted tooth. FKBP5 displayed minimal levels in the pulp tissue of both healthy and carious human teeth, with no significant difference observed. In human pulpitis, FKBP5 expression was significantly elevated compared to normal and carious teeth (*P* < .01) and detected in the odontoblast layer (Figure 2B). In the rat pulp injury model, hematoxylin and eosin (HE) staining revealed significant infiltration of inflammatory cells in the pulp, with increasing inflammatory damage over time (Figure 2C). FKBP5 expression initially increased before decreasing: FKBP5 expression was upregulated at 3 h after pulp injury, remaining high from 12 h to 3 days, returning to the initial level by 7 days (Figure 2D). In the *in vitro* model, FKBP5 was significantly elevated in DPSCs after OD induction (Figure 2E and F). *FKBP5* expression was significantly upregulated at 14 days, consistent with RNA-seq data suggesting a peak in DEGs at this time point. Thus, we used 14 days for developing an *in vitro* OD model. After 3 h of LPS stimulation, no significant change in FKBP5 protein expression was observed compared to the control group, despite a modest elevation of FKBP5 expression after 1 day (Figure 2G).

### Role of FKBP5 in dentin formation and *in vitro* and *in vivo* anti-inflammatory effects

**Figure 3:**
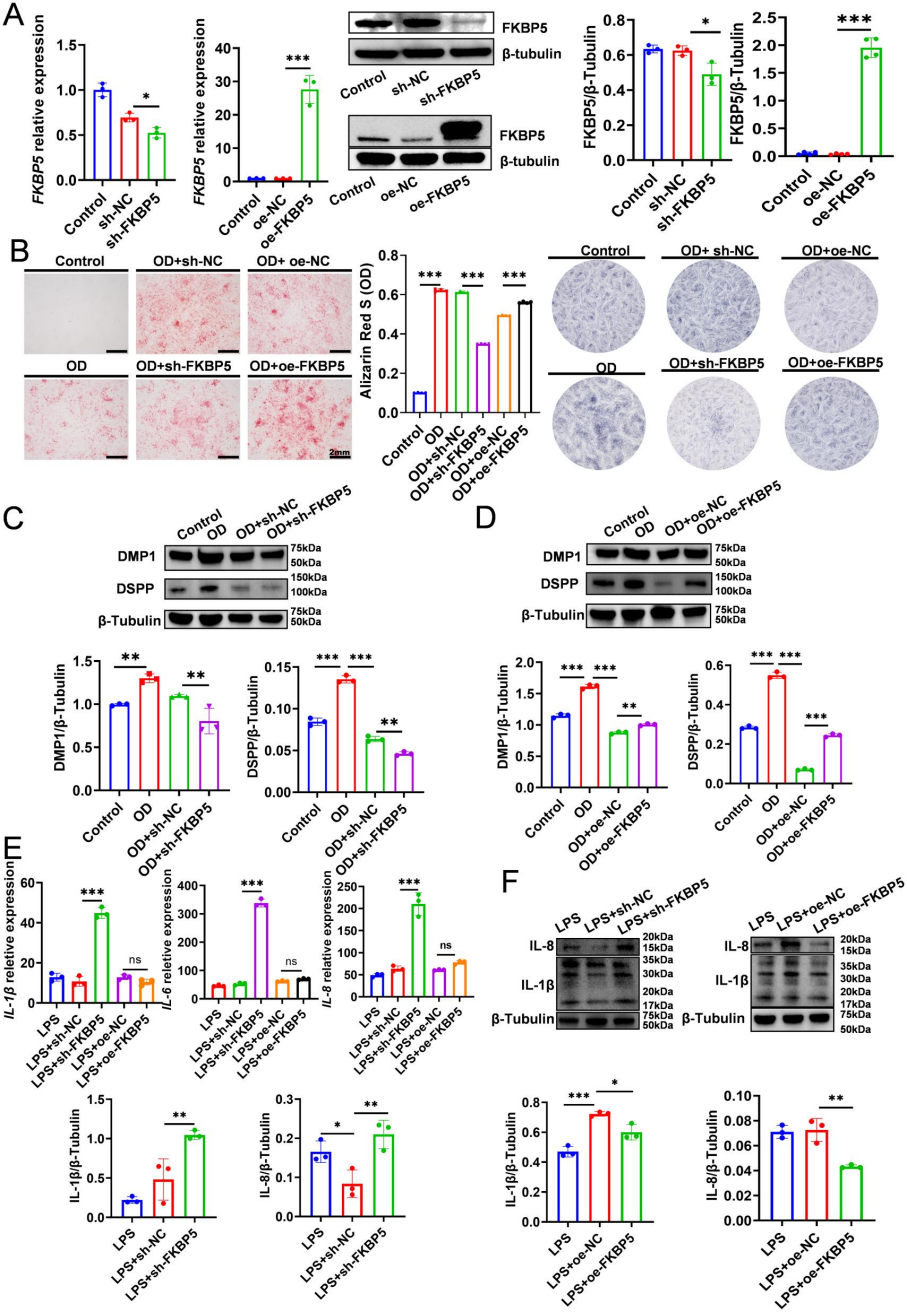
The effect of FKBP5 knockdown and overexpression on dentin formation (mineralization) *in vitro*. (A) DPSCs with FKBP5 knockdown or overexpression were analyzed using qPCR and Western blot assays (*N *= 3; *P < .05; ***P < .001). (B) Alizarin red staining was conducted after 21 days of *in vitro* OD induction in DPSCs with FKBP5 knockdown/overexpression, accompanied by calcium ion concentration analysis (*N *= 4, ***P < .001), and alkaline phosphatase (ALP) staining after 7 days of OD induction. (C and D) The detection of DMP-1 and DSPP proteins after 14 days of *in vitro* OD induction (*N *= 3, **P < .01; ***P < .001); (E) The gene expressions of IL-1β, IL-6, and IL-8 after stimulation of LPS for 24 h (*N *= 3, ***P < .001). (F) The protein levels of IL-1β, IL-6, and IL-8 after stimulation of LPS for 24 h (*N *= 3, *P < .05; **P < .01; ***P < .001).

DPSCs with low or high FKBP5 expression were used to examine their role in dentin formation (mineralization) *in vitro*. The sh-FKBP5 group exhibited lower FKBP5 expression at gene and protein levels than the negative control (sh-NC), whereas the oe-FKBP5 group demonstrated increased gene and protein expression relative to the negative control (oe-NC; Figure 3A). After 21 days of OD induction, alizarin red staining and calcium ion concentration analysis revealed a greater formation of mineralized nodules in the OD+oe-FKBP5 group than in the OD+oe-NC group, whereas the OD+sh-FKBP5 group exhibited a significant reduction in mineralized nodules relative to the OD+sh-NC group. The calcium ion concentrations yielded similar results. After 7 days of OD induction, alkaline phosphatase (ALP) staining revealed a notable reduction in ALP activity in OD+sh-FKBP5 relative to OD+sh-NC and an increase in ALP activity in OD+oe-FKBP5 compared to OD+oe-NC (Figure 3B). Dentine sialophosphoprotein (DSPP) and DMP-1 were examined at 14 days after OD induction. The results indicated a significant reduction in the number of mineralized nodules in the OD+sh-NC group relative to the OD+sh-FKBP5 group (Figure 3C). Additionally, DSPP and DMP-1 expression was markedly downregulated in the OD+sh-FKBP5 group compared to the OD+oe-FKBP5 group but significantly upregulated in the OD+oe-FKBP5 group compared to the OD+oe-NC group (Figure 3D). FKBP5 has the potential to regulate reparative dentin formation by increasing odontogenic differentiation *in vitro*.

Next, we stimulated DPSCs with LPS to examine their impact on the inflammatory response. After 24 h of LPS stimulation, IL-1β and IL-8 expression were markedly elevated in the LPS+sh-FKBP5 group relative to the LPS+sh-NC group. In the LPS+oe-FKBP5 group, *IL-1β* and *IL-8* expression did not exhibit significant changes compared to LPS+oe-NC, but their protein levels were markedly downregulated. FKBP5 may exert a negative regulatory influence on the inflammatory response (Figure 3E and F).

**Figure 4:**
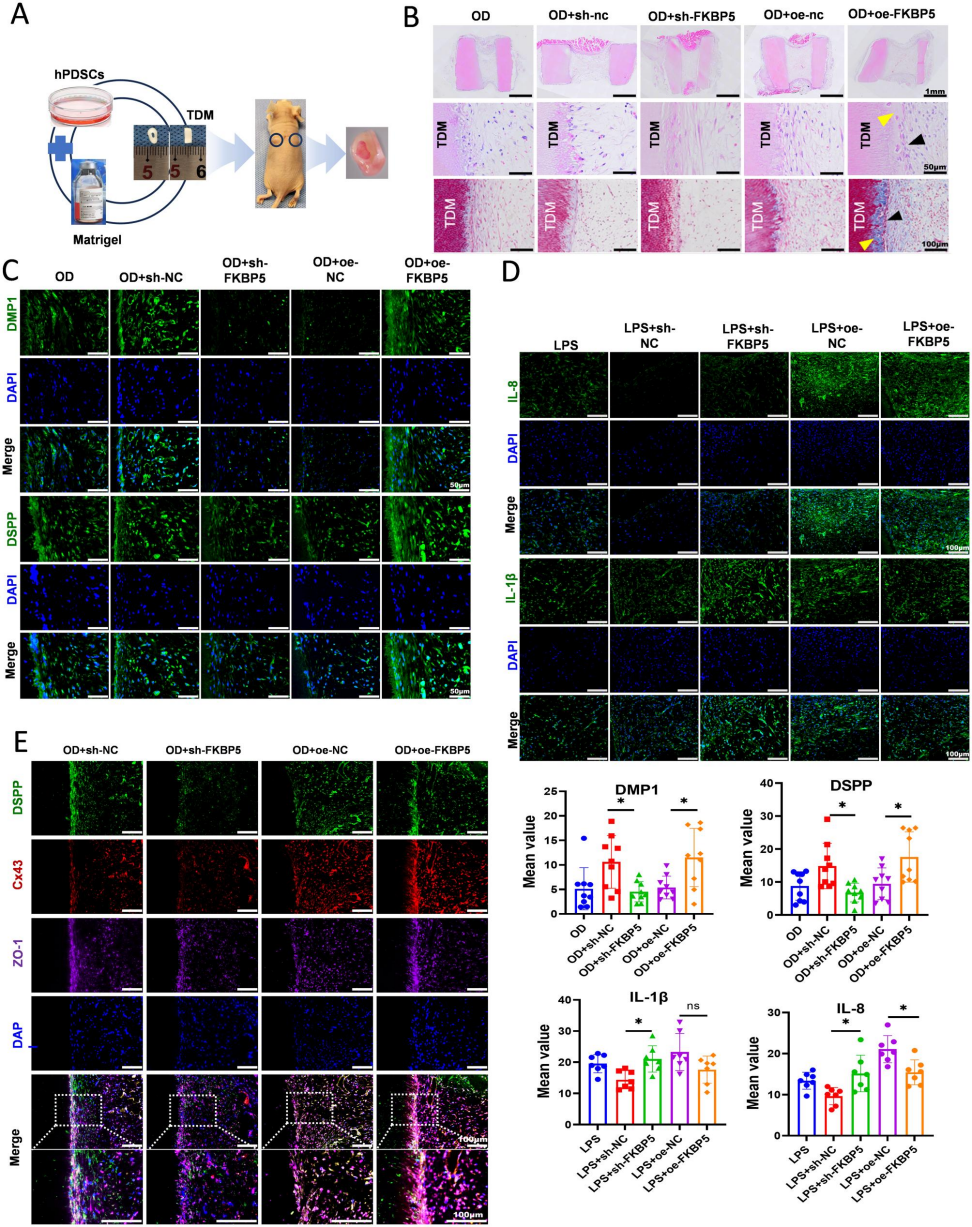
The impact of FKBP5 knockdown/overexpression on dentin formation *in vivo*. (A) Schematic illustration of the *in vivo* ectopic model of TDM in the dorsal subcutis of nude mice. (B) HE staining and Masson staining in the *in vivo* model generated by OD induction. (C) Expression levels of DMP-1 and DSPP in the *in vivo* model caused by OD induction in DPSCs with FKBP5 knockdown/overexpression for 6 weeks (*N *= 9, *P < .05). (D) Expression levels of IL-1β and IL-8 in the *in vivo* model caused by LPS stimulation in DPSCs with FKBP5 knockdown/overexpression for 1 week (*N *= 7, ns, *P *> .05; *P < .05). (E) Co-expression of DSPP, Cx43, and ZO-1 using multiple immunofluorescence labeling in the *in vivo* model after OD induction.

To assess *in vivo* effects in dentin formation, we developed an OD model by transplanting DPSCs with a human-TDM composite into the dorsal subcutis of nude mice (Figure 4A). Six weeks post-transplantation, FKBP5 protein expression was markedly downregulated in the knockdown group and upregulated in the overexpression group, indicating successful establishment of an *in vivo* model of FKBP5 knockdown/overexpression (Figure S2). HE and Masson staining revealed that the OD+oe-FKBP5 group developed a more compact synthetic odontoblast-like cellular layer and dentin-like layer in the proximal dentin region (Figure 4B). Immunofluorescence (IF) staining indicated decreased DSPP and DMP-1 in the OD+sh-FKBP5 group relative to the OD+NC group but increased with FKBP5 overexpression (Figure 4C). The odontoblast-like cellular layer exhibited positive expression and colocalization of DSPP, gap junction protein Cx43, and Zonula Occludens-1 (ZO-1). The expression of these proteins was reduced with FKBP5 knockdown and increased after overexpression. A distinct odontoblast-like cellular layer was identified in the OD+oe-FKBP5 group but not in the OD+sh-FKBP5 and OD+oe-NC groups (Figure 4E). To clarify the role of FKBP5 in pulp inflammation, we developed an *in vivo* model of inflammation triggered by LPS. One week post-transplantation, IL-1β and IL-8 expression were elevated in the LPS+sh-FKBP5 group compared to the LPS+sh-NC group. Conversely, FKBP5 overexpression decreased IL-8 expression, with no significant variation in IL-1β expression when compared to the LPS+oe-NC group (Figure 4D). FKBP5 promoted reparative dentin formation by increasing odontogenic differentiation and alleviating inflammation *in vivo*.

### H3K9ac and H3K27ac regulate FKBP5 expression in DPSCs during OD induction

**Figure 5:**
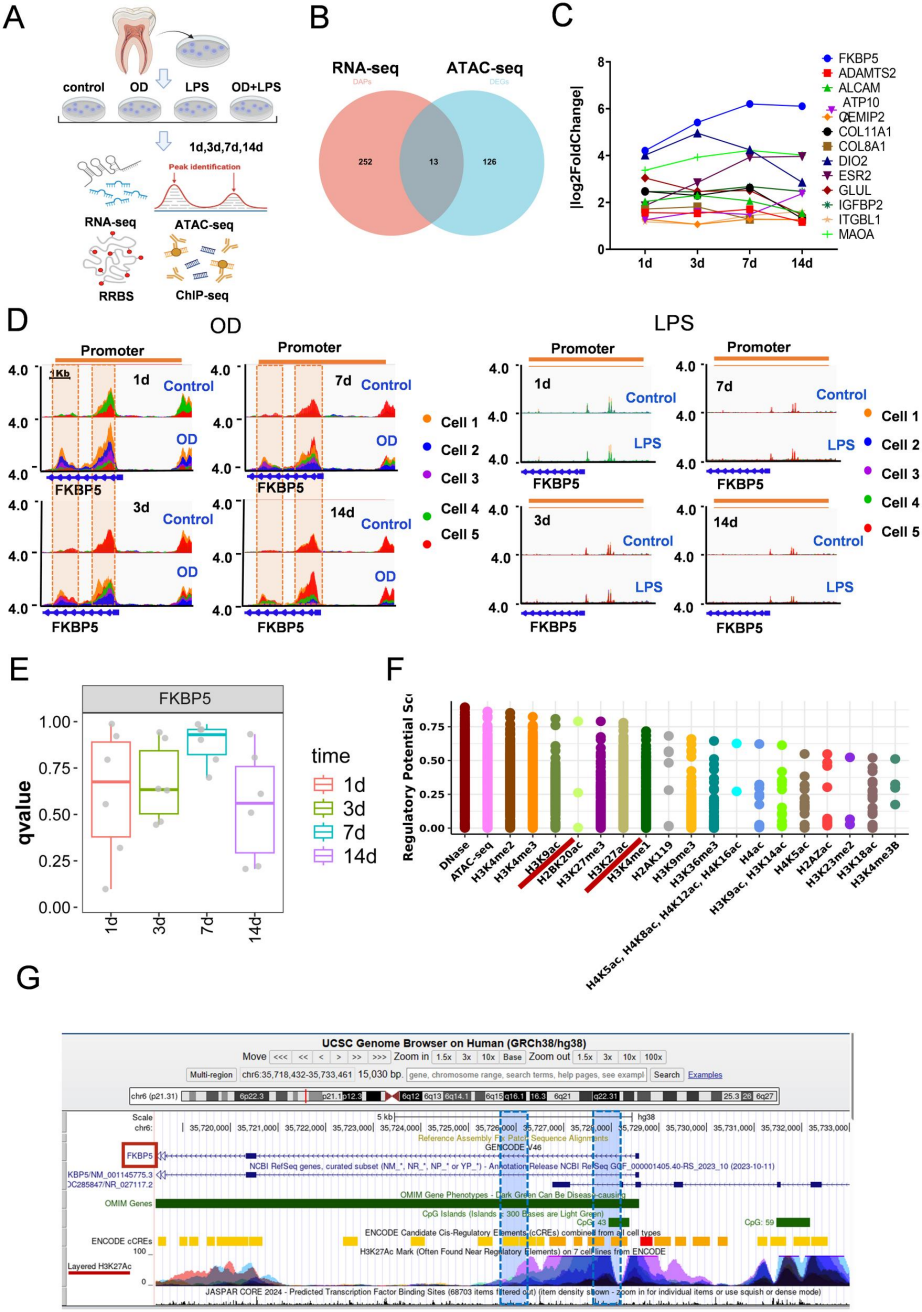
Integrated multi-omics examination of the FKBP5 chromatin remodeling process. (A) Schematic illustration of the integrated multi-omics analysis of DPSCs under OD and LPS stimulation. (B) Intersection of DEGs from RNA-seq and DAPs from ATAC-seq identified 13 candidate genes. (C) Differential expression of the 13 candidate genes under OD induction at four time points, 1, 3, 7, and 14 days. (D) IGV visualization of the FKBP5 promoter region of five DPSCs at the four time points, by ATAC-seq. E. The methylation map of the FKBP5 promoter region by RRBS sequencing (*N *= 6). (F) The Cistrome Data Browser database predicts FKBP5’s involvement in the regulation of histone methylation and acetylation. (G) UCSC visualization of the FKBP5 promoter region. The blue box denotes the positions of two markedly distinct accessible peaks within the potential promoter region of FKBP5, as revealed by the ATAC-seq; the peak signals, depicted in various colors in the figure, correspond to H3K27ac modifications across different tissues or cell types from ENCODE or Roadmap Epigenomics, respectively.

What mechanism regulates FKBP5 expression in DPSCs during OD induction? We conducted an integrated analysis of RNA-seq and ATAC-seq at various time intervals (1, 3, 7, and 14 days) (Figure 5A). ATAC-seq data were examined for differentially accessible peaks (DAPs). The intersection of the DAPs from the four time points resulted in a total of 265 DAPs. The 139 DEGs from the RNA-seq analysis and 265 DAPs from the ATAC-seq analysis were intersected, resulting in the identification of 13 genes (Figure 5B). The fold change of FKBP5 was the highest at all time intervals and exhibited a consistent upregulated trend (Figure 5C). Analysis of ATAC-seq from the FKBP5 promoter region, as seen by Integrative Genomics Viewer, indicated that the intensity of the two peak signals around the transcriptional start site of the FKBP5 gene exhibited an increase in the OD group compared to the control group (*P *< .05, *N* = 5). Under LPS stimulation, the chromatin accessibility of the FKBP5 promoter region exhibited no significant alterations in accessible peaks at the four time periods compared to the control group (Figure 5D). Chromatin remodeling, a crucial mechanism regulating chromatin accessibility, may affect the expression of FKBP5 by modifying its promoter region.

What mechanisms facilitate the chromatin remodeling of FKBP5? Epigenetic modification may potentially dominate chromatin remodeling. DNA methylation in the promoter region of FKBP5 was detected by reduced representation bisulfite sequencing (RRBS), and no significant variation was found in the corrected *P*-value (q-value) across the four time periods after OD induction, suggesting that DNA methylation was not involved in the OD process (Figure 5E). Given that histone acetylation typically facilitates transcription, we hypothesized the involvement of histone modifications in chromatin remodeling within the FKBP5 promoter region. The results revealed a strong correlation between FKBP5 and both methylation and acetylation modifications of histones, as indicated by data from the Chromatin Immunoprecipitation with High-throughput Sequencing (ChIP-seq) public database (Figure 5F). Notably, H3K9ac, H2BK20ac, and H3K27ac were among the highest-ranked modifications, with H3K9ac and H3K27ac being more extensively documented. The ATAC-seq data track of the UCSC Genome Browser demonstrates substantial overlap of peak signals from two differentially accessible regions near the FKBP5 gene promoter with multiple peaks in the layered H3K27ac row, suggesting that the FKBP5 promoter region may be modulated by H3K27ac during OD induction (Figure 5G). ChIP-seq assay revealed that H3K9ac and H3K27ac levels were increased in the promoter region of FKBP5 after 7 days of OD induction (Figure S3).

### Enhances histone acetylation regulation of FKBP5 and reparative dentin formation

**Figure 6:**
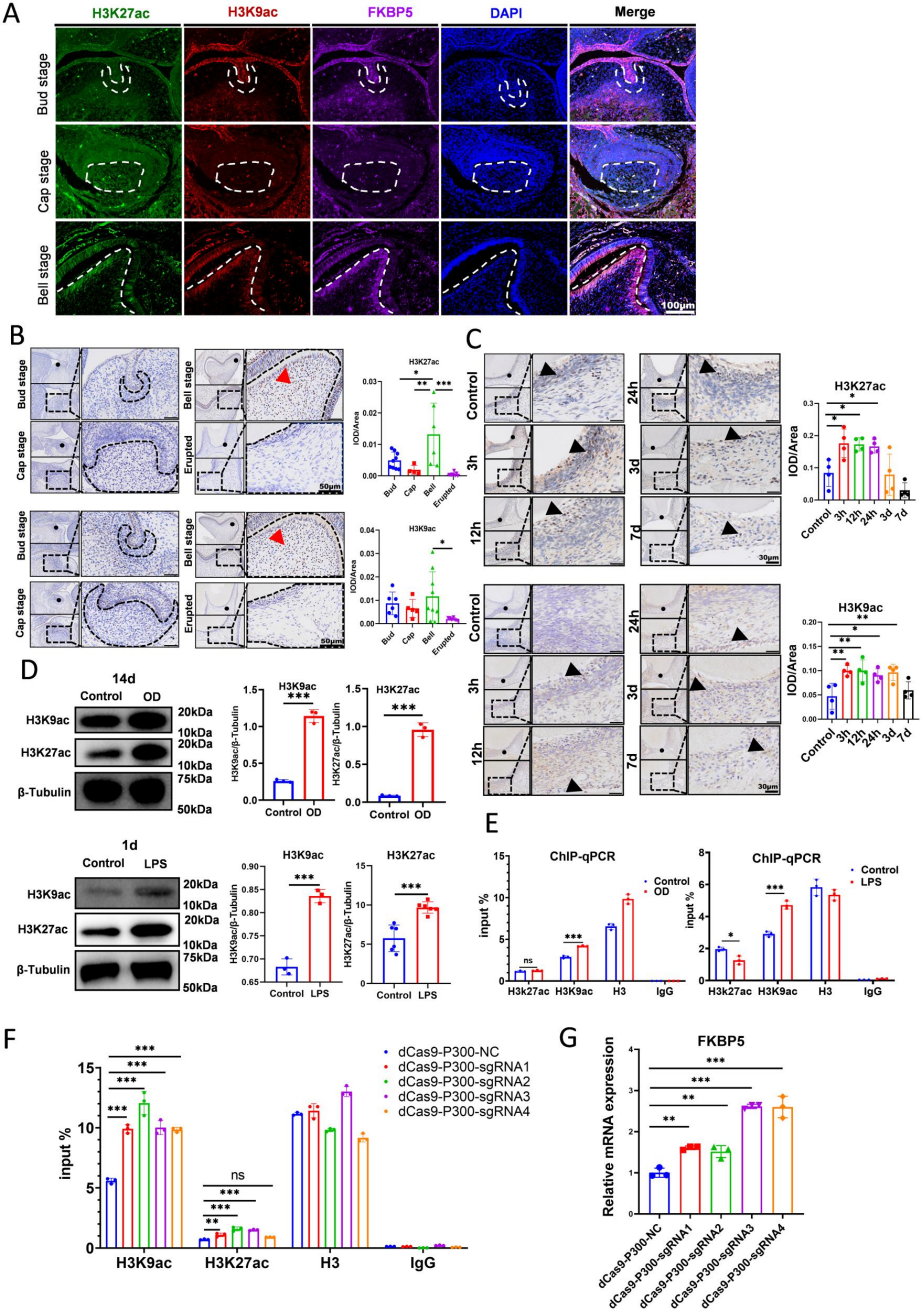
Histone acetylation at H3K9ac and H3K27ac participates in dentin formation and FKBP5 expression. (A) Staining of H3K9ac and H3K27ac in rat tooth germ. (B) IHC staining and quantitative analysis of H3K9ac and H3K27ac (*N *= 4–9, *P < .05; **P < .01; ***P < .001). (C) Levels of acetylated histones H3K27ac and H3K9ac in a rat pulp injury model (*N *= 4, *P < .05; **P < .01). (D) Expression of H3K9ac and H3K27ac in DPSCs following OD induction and LPS stimulation *in vitro* (*N *= 3–6, ***P < .001). (E) Enrichment of H3K27ac and H3K9ac in the FKBP5 promoter region was assessed by ChIP-qPCR in DPSCs subjected to OD induction and LPS stimulation (*N *= 3, ns, *P *> .05; *P < .05; ***P < .001). (F) ChIP-qPCR indicated that following the application of the dCas9-P300 system, the FKBP5 promoter exhibited enrichment for acetylated histones H3K9ac and H3K27ac (*N *= 3, ns, *P *> .05; **P < .01; ***P < .001). H3: Positive control; IgG: Negative control; (G) qPCR assessment of FKBP5 mRNA levels after epigenetic modification of dCas9-P300.

The results initially validated that H3K9ac and H3K27ac may be linked to FKBP5 chromatin remodeling, which we subsequently corroborated. IF staining of rat tooth germ at various stages demonstrated high expression of H3K27ac, H3K9ac, and FKBP5 in the dentin layer, co-localizing at the bell stage of intensive dentin deposition (Figure 6A). During various developmental phases of the rat tooth germ, the expression of histone acetylation changes was minimal at the cap and bud stages, peaking at the bell stage (Figure 6B). This expression pattern was markedly consistent with FKBP5 expression throughout rat odontogenesis. In the rat pulp injury model, H3K27ac was strongly expressed and downregulated to control levels by the third day, but the increased H3K9ac expression decreased to control levels by the seventh day (Figure 6C). After 14 days of OD induction, H3K9ac and H3K27ac expression were significantly elevated, as shown by Western blot analysis. H3K27ac and H3K9ac expression were elevated after 1 day of LPS stimulation in DPSCs (Figure 6D). Next, we assessed the H3K9ac and H3K27ac enrichment in the FKBP5 promoter area using ChIP-qPCR. OD induction increased the presence of H3K9ac in the promoter region of FKBP5, but did not affect H3K27ac. H3K9ac enrichment in the FKBP5 promoter area similarly increased after LPS stimulation, whereas that of H3K27ac was reduced (Figure 6E). Based on ATAC-seq analysis, we synthesized particular sgRNAs targeting the two differential peaks within the FKBP5 promoter region (Figure S4). We cotransfected dCas9-p300 lentivirus and sgRNA lentivirus in DPSCs to determine the effect of histone acetylation on the transcriptional regulation of FKBP5. In comparison to the control group, the dCas9-p300-sgRNA systems increased H3K9ac and H3K27ac presence in the FKBP5 promoter region. Accordingly (Figure 6F), significant upregulation in FKBP5 mRNA expression was observed (Figure 6G).

**Figure 7:**
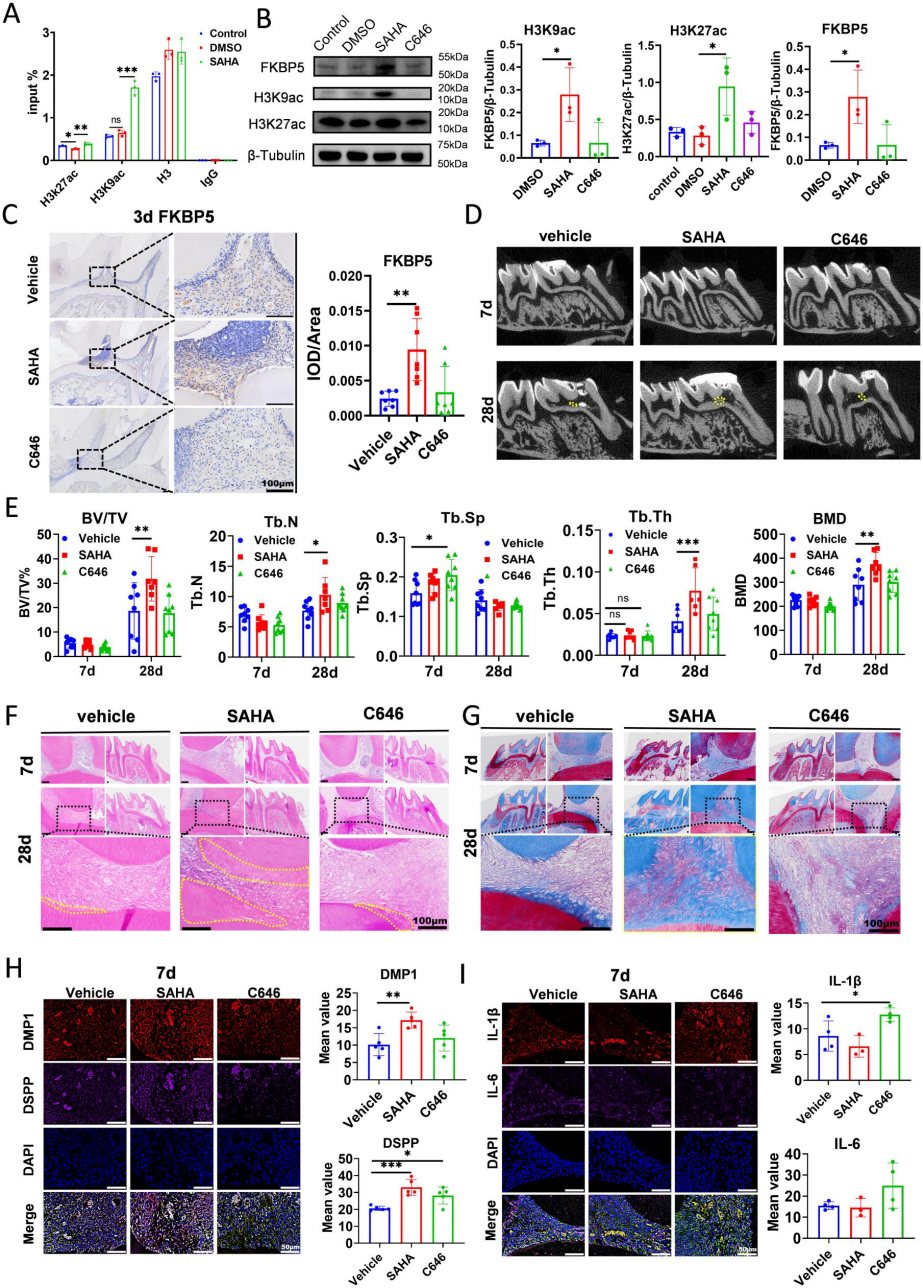
Enhanced histone acetylation promotes FKBP5 expression and dentin barrier formation. (A) Enrichment of H3K27ac and H3K9ac in the promoter region of FKBP5 in DPSCs after enhanced histone acetylation (*N *= 3, *P < .05; **P < .01; *** *P *< .001). (B) Effect of SAHA and C646 on the expression of FKBP5, H3K9ac, and H3K27ac in DPSCs (*N *= 3, *P < .05). (C) IHC staining in the rat pulp injury model showing the effect of SAHA and C646 on the expressions of FKBP5 (*N *= 7, **P < .01). (D) Micro-CT showed mineralized tissue in the pulp cavity. (E) Micro-CT parameters, BV/TV, Tb.N, Tb.Th, Tb.Sp, and BMD of mineralized tissue in the pulp cavity (*N *= 7–8, *P < .05; **P < .01; ***P < .001). (F) HE staining showing dentin barrier. (G) Masson staining showing collagen formation in newly formed dentin. (H) Multiple immunofluorescent staining of DMP-1 and DSPP in rat pulp injury model of 7 days (*N *= 5, *P < .05; **P < .01; *** *P *< .001). (I). Immunofluorescence staining and quantitative analysis of IL-1β and IL-6 in a rat pulp injury model of 7 days (*N *= 3–4, *P < .05).

Subsequently, we employed the HDAC inhibitor Vorinostat (SAHA) and the p300/CBP-specific inhibitor C646 to modulate histone acetylation levels. ChIP-qPCR demonstrated increased binding of both H3K27ac and H3K9ac at the FKBP5 promoter region in DPSCs after SAHA stimulation (Figure 7A). Western blot analysis demonstrated that SAHA markedly increased H3K27ac and H3K9ac protein levels, together with upregulation of FKBP5 expression, while no significant changes were detected in the C646 group (Figure 7B). In the rat pulp injury model, SAHA significantly increased FKBP5 expression compared to the vehicle group at three days, whereas C646 had no effect on FKBP5 expression (Figure 7C).

Micro-CT data revealed no significant density in the pulp cavity on day 7; nevertheless, on day 28, substantially high-density images resembling reparative dentin appeared in the pulp cavity near the injury site of pulp (Figure 7D). On day 7, there were no notable alterations in bone mineral density (BMD), bone volume/total volume (BV/TV), trabecular thickness (Tb. Th), and trabecular number (Tb. N) in the SAHA group compared to the vehicle group. However, the C646 group had a significantly increased Tb. Sp. At 28 days, BV/TV, Tb. N, Tb. Th, and BMD were elevated in the SAHA group compared to the vehicle group. No notable alterations were detected in any parameters for the C646 group (Figure 7E). HE staining demonstrated no evident dentin formation after seven days. However, at 28 days, the emergence of a reparative dentin with a well-defined boundary was observed. Masson staining demonstrated collagen formation in newly formed dentin, more evident in the SAHA-treated group, leading to a reparative dentin structure (Figure 7F and G). During 7 days, SAHA increased DMP-1 and DSPP expression, whereas C646 suppressed DMP-1 expression and had no significant impact on DSPP (Figure 7H). At 28 days, SAHA still increased DMP-1 and DSPP expression, but C646 did not influence either (Figure S7B). After seven days, SAHA suppressed IL-1β expression but not IL-6, whereas C646 did not significantly modulate either cytokine (Figure 7I). The results after 3 days exhibited a consistent trend (Figure S7A). Thus, SAHA appears to contribute to the mineralization and inflammatory response of DPSCs by augmenting histone acetylation and modulating FKBP5 expression to facilitate the development of a nascent reparative dentin, whereas C646 has a little regulatory effect on FKBP5.

## Discussion

The dentin bridge acts as a shield, preventing further irritation or bacterial contamination by keeping the exposed pulp tissue from the oral environment. Among the clinical successes of pulp capping are radiographic success, the creation of dentin bridges, and the thickness of dentin bridges. For both direct and indirect pulp capping, as well as for VPT, the successful construction of a dentin bridge was necessary in clinical practice. Achieving a solid reparative barrier (dentin barrier) is of the utmost importance since it not only acts as a protective shield for the pulp that lies beneath it, but it also implies that the treatment has been successful in evoking a biological reaction.

Reparative dentin refers to dentin formed by odontoblast-like cells differentiated from undifferentiated stem cells within the dental pulp under severe acute stimulation, after death of the original odontoblasts.^23^ The development of reparative dentin is a multifaceted biological process that comprises cellular differentiation, signaling, and matrix deposition. Their synergistic activities safeguard and rejuvenate the pulp.^24^ Formation of reparative dentin occurs through the synthesis of a collagenous matrix, predominantly consisting of type-I collagen and fibronectin, which establishes the structural framework necessary for mineralization.^25^^,^^26^ The subsequent mineralization is due to calcium and phosphorus deposition,^27^ leading to reparative odontogenesis, which aids in the repair and protection of the dental pulp. Wnt/β-catenin signaling pathways regulate DPSC proliferation and differentiation, augmenting tissue repair and regeneration. Wnt ligands, such as Wnt3a, facilitate reparative dentin formation by enhancing Osterix, Bone Morphogenetic Protein 2, and DMP-1 expression.^28^ Small Integrin Binding Ligand N-linked Glycoproteins, such as DSPP and DMP-1, facilitate dentine healing and regulate odontoblast differentiation.^29^^,^^30^ Matrix metalloproteinases and their inhibitors meticulously regulate extracellular matrix remodeling, releasing, and activating latent growth factors to maintain tissue turnover.

The aforementioned signaling pathways and factors participate in reparative (tertiary) dentin development. Dentin bridges and reparative dentin are interfaces that directly engage with the damage site and encompass several pathological and physiological processes. Two prerequisites should be satisfied for the formation of reparative dentin: to avert pulp necrosis and turn inflammation toward beneficial ways to facilitate tissue repair^31^^,^^32^ and to ensure contact between the pulp-capping material and pulp stumps stimulates odontoblastic activity, leading to dentine bridge formation.^14^ Current research on promoting reparative dentin formation mostly focuses on the effects of pulp-capping materials but lacks investigations into the underlying mechanisms.^10^^,^^33^^,^^34^ DPSCs are an important source of newly formed odontoblast-like cells following pulp injury.^35^^,^^36^ Therefore, we investigated reparative dentin formation by OD induction *in vitro* and established a pulp injury model *in vivo*. RNA-seq of DPSCs showed elevated FKBP5 expression during OD, implying a potential role in OD induction. Nevertheless, FKBP5 expression remained unaffected by LPS stimulation, thus showing that pulp inflammatory stimuli are unlikely to elevate FKBP5 expression.

FKBP5 encodes for the Hsp90-associated cochaperone FK506 binding protein 51 (FKBP51), a down-regulator of stress signaling during acute stress. Similarly, FKBP5 is involved in inflammation-related diseases. For example, under aging and stress, epigenetic upregulation of FKBP5 promotes NF-κB-driven inflammation and increases the risk of cardiovascular disease.^37^ Recent findings indicate that FKBP5 significantly contributes to cell differentiation. A study reported that FKBP5 enhances the osteogenesis of human adipose-derived stem cells into osteocytes.^38^ Increased FKBP5 expression accelerated osteogenesis of mesenchymal stem cells by inhibiting the type-I IFN response.^39^ These results indicated that FKBP5 could mitigate immune-related bone loss and enhance bone regeneration. Further, FKBP5 is a positive regulator of myoblast differentiation and muscle regeneration.^40^^,^^41^ However, its involvement in dental formation or odontogenesis has not been reported until now. This study demonstrated upregulated FKBP5 expression during tooth germ development and pulp repair. FKBP5 knockdown or overexpression influenced the odontoblastic differentiation of DPSCs. However, FKBP5 overexpression did not fully reverse the downregulation of DMP-1, DSPP, and IL-1β induced by FKBP5 knockdown. This may be related to intracellular homeostatic constraints,^42^^,^^43^ the dependence of FKBP5 on cooperating molecules such as HSP90 and GR,^44^^,^^45^ and the fact that DMP-1 and DSPP are coregulated by multiple signaling pathways.^46^  *In situ* transplantation revealed that FKBP5 overexpression affected dentin formation, where a fenestrated configuration of odontoblast-like structures and synthesis of dentin and newly formed collagen were observed. This indicates that FKBP5 is a crucial factor in promoting reparative dentin formation and plays a significant role in collagen synthesis (Masson staining) and mineralization (DSPP, DMP-1 expression, and calcium deposition) of tertiary dentin. The regulation of inflammation by FKBP5 is markedly cell type-dependent and may exhibit either pro-inflammatory^37^^,^^47^ or anti-inflammatory effects.^48^^,^^49^ In immune cells, such as monocytes/macrophages, FKBP5 promotes inflammation through NF-κB activation,^37^ and in neural cells, it facilitates neuroinflammation.^47^ In contrast, FKBP5 downregulates the IFN-γ and IL-17 in T lymphocytes,^49^ and suppresses IL-6 secretion in trophoblasts.^48^ However, in the present study, the regulatory effect of FKBP5 on inflammation was limited to the expression of inflammatory factors, and the underlying mechanism remains to be further investigated together with classical pathways such as NF-κB, MAPK, and glucocorticoid signaling.^37^^,^^45^

By what mechanism do DPSCs upregulate FKBP5 expression? The two peak signals at the FKBP5 gene promoter under OD induction exhibited an upward trend compared to the control group. However, no significant change was observed with LPS stimulation. Chromatin remodeling of FKBP5 may contribute to its upregulation. A potential epigenetic modification in FKBP5 is DNA methylation, frequently linked to stress-induced psychological effects. Early-life stress was reported to influence this FKBP5 function via DNA demethylation.^50^^,^^51^ Genetic and epigenetic variations in FKBP5 have been consistently linked to the onset of stress-related mental illnesses, including depression, anxiety disorders, and posttraumatic stress disorder.^52–54^ We conducted RRBS sequencing to assess DNA methylation and demonstrated that FKBP5 levels did not exhibit significant differences under OD induction compared to control. Thus, DNA methylation may not be linked to the differential effect of FKBP5.

Histone acetylation of *FKBP5* is scarcely reported. We examined ChIP-seq data in the UCSC Genome Browser, where the cervical cancer cell line, human embryonic stem cell, and human fetal lung fibroblast cell line demonstrated the existence of H3K27ac modifications in the promoter region of FKBP5. ATAC-seq of the OD group revealed increased accessibility signals in the FKBP5 promoter region, coinciding with the H3K27ac modification in the UCSC Genome Browser database. This indicates a potential role of histone acetylation in the pro-differentiation effect of FKBP5. Moreover, ChIP-seq and ChIP-PCR findings validated the association between histone acetylation alteration and the pro-differentiation effect of FKBP5. ChIP-seq and ChIP-qPCR further confirmed that changes in histone acetylation were strongly related to *FKBP5* overexpression. Can epigenetic regulation augment the expression of FKBP5 and promote reparative dentin formation?

Epigenetic regulation influences gene transcription outcomes without modifying the gene sequence and is both reversible and personalized. To address whether histone acetylation facilitates reparative dentin formation via FKBP5, we conducted pan-enhanced acetylation with SAHA, a broad-spectrum HDAC inhibitor targeting class I and class II HDACs, which promotes the upregulation of H3K9ac and H3K27ac. The findings indicated a decreased necrotic area of the pulp after 28 days, accompanied by the development of a layer of newly formed dentin between the necrotic zone and the pulp. Micro-CT detection indicated mineral deposition in the region, implying the development of reparative dentin. Increased FKBP5 expression was observed in the same region, confirming the significant involvement of FKBP5 in reparative dentin development. Odontoblast differentiation was confirmed by detecting DMP-1 and DSPP, while IL-1β and IL-6 signaled a suppressive inflammatory reaction. The dual mechanism is aligned with the physiological process of reparative dentin formation. Our group previously reported that the modulation of histone acetylation facilitates coordinated healing of soft and hard tissues within the pulp-dentin complex.^22^ FKBP5 may facilitate reparative dentin formation, whereas NRG1 may enhance pulp healing, vascularization, and neural repair, with both contributing synergistically to the comprehensive restoration of soft and hard tissues. These key targets are involved in the repair process following pulp injury and may serve as potential molecular targets for optimizing direct pulp capping in the future, thereby improving treatment success rates.

## Conclusion

FKBP5 facilitated reparative dentin formation and neoplastic collagen synthesis at the injury site while mitigating inflammation. Histone acetylation was the mechanism that modulated the odontogenic differentiation effects of FKBP5. SAHA-mediated acetylation enhances reparative dentin synthesis at the injury site via FKBP5 activity.

## Supplementary Material

szag047_Supplementary_Data

## Data Availability

The data supporting the findings of this study are available from the corresponding author upon reasonable request.

## References

[1] Delgado-Pérez VJ , Patiño-Marín N, Rueda-Ibarra V, et al Epidemiological and oral public health aspects of dental pain: a narrative review. Cureus. 2024;16:e74908. 10.7759/cureus.74908 39742195 PMC11687405

[2] Santos PS , Barasuol JC, Moccelini BS, et al Prevalence of toothache and associated factors in children and adolescents: a systematic review and meta-analysis. Clin Oral Investig. 2022;26:1105-1119. 10.1007/s00784-021-04255-2 34791550

[3] Siqueira JF Jr. , Rôças IN. Present status and future directions: microbiology of endodontic infections. Int Endod J. 2022;55:512-530. 10.1111/iej.13677 34958494

[4] Van Nieuwenhuysen JP , D’Hoore W, Leprince JG. What ultimately matters in root canal treatment success and tooth preservation: a 25-year cohort study. Int Endod J. 2023;56:544-557. 10.1111/iej.13895 36683563

[5] Nygaard-Ostby B. [Mortal or vital treatment of the inflamed pulp?]. SSO Schweiz Monatsschr Zahnheilkd. 1966;76:545-551. 5220540

[6] Tomson PL , Vilela Bastos J, Jacimovic J, Jakovljevic A, Pulikkotil SJ, Nagendrababu V. Effectiveness of pulpotomy compared with root canal treatment in managing non-traumatic pulpitis associated with spontaneous pain: a systematic review and meta-analysis. Int Endod J. 2023;56:355-369. 10.1111/iej.13844 36209498

[7] Akhil VS , Kumar V, Aravind A, et al Novel cryotherapy technique for pulpotomy in mature permanent teeth with symptomatic irreversible pulpitis- a randomized controlled trial. Clin Oral Investig. 2024;28:275. 10.1007/s00784-024-05661-y 38668793

[8] Taha NA , Abuzaid AM, Khader YS. A randomized controlled clinical trial of pulpotomy versus root canal therapy in mature teeth with irreversible pulpitis: outcome, quality of life, and patients’ satisfaction. J Endod. 2023;49:624-631.e2. 10.1016/j.joen.2023.04.001 37080387

[9] Darwish SS , Abd El Meguid SH, Wahba NA, Mohamed AA, Chrzanowski W, Abou Neel EA. Root maturation and dentin-pulp response to enamel matrix derivative in pulpotomized permanent teeth. J Tissue Eng. 2014;5:2041731414521707. 10.1177/2041731414521707 24551447 PMC3924881

[10] Silva E , Pinto KP, Riche F, et al A meta-analysis of calcium silicate-based cements and calcium hydroxide as promoters of hard tissue bridge formation. Int Endod J. 2025;58:685-714. 10.1111/iej.14210 39988950

[11] Baranwal HC , Mittal N, Yadav J, Rani P, Naveen Kumar PG. Outcome of partial pulpotomy verses full pulpotomy using biodentine in vital mature permanent molar with clinical symptoms indicative of irreversible pulpitis: a randomized clinical trial. J Conserv Dent. 2022;25:317-323. 10.4103/jcd.jcd_118_22 35836550 PMC9274703

[12] Devi TP , Kaur A, Priyadarshini S, Deepak BS, Banerjee S, Sanjeeta N. Histological evaluation of dental pulp response to biodentine, enamel matrix derivative (emdogain), and mineral trioxide aggregate as direct pulp-capping agents – a randomized clinical trial. 2023;37:107-112. 10.4103/jms.jms_26_23

[13] Bakhtiar H , Nekoofar MH, Aminishakib P, et al Human pulp responses to partial pulpotomy treatment with TheraCal as compared with biodentine and ProRoot MTA: a clinical trial. J Endod. 2017;43:1786-1791. 10.1016/j.joen.2017.06.025 28822566

[14] Goldberg M , Njeh A, Uzunoglu E. Is pulp inflammation a prerequisite for pulp healing and regeneration? Mediators Inflamm. 2015;2015:347649. 10.1155/2015/347649 26538825 PMC4619968

[15] Cooper PR , Takahashi Y, Graham LW, Simon S, Imazato S, Smith AJ. Inflammation-regeneration interplay in the dentine-pulp complex. J Dent. 2010;38:687-697. 10.1016/j.jdent.2010.05.016 20580768

[16] Farges JC , Alliot-Licht B, Renard E, et al Dental pulp defence and repair mechanisms in dental caries. Mediators Inflamm. 2015;2015:230251. 10.1155/2015/230251 26538821 PMC4619960

[17] Finkelman RD , Mohan S, Jennings JC, Taylor AK, Jepsen S, Baylink DJ. Quantitation of growth factors IGF-I, SGF/IGF-II, and TGF-beta in human dentin. J Bone Mine Res 1990;5:717-723. 10.1002/jbmr.5650050708 2396498

[18] He G , Dahl T, Veis A, George A. Nucleation of apatite crystals *in vitro* by self-assembled dentin matrix protein 1. Nat Mater. 2003;2:552-558. 10.1038/nmat945 12872163

[19] Shah D , Lynd T, Ho D, et al Pulp–Dentin tissue healing response: A discussion of current biomedical approaches. JCM. 2020;9:434. 10.3390/jcm9020434 32033375 PMC7074340

[20] Bennett RL , Licht JD. Targeting epigenetics in cancer. Annu Rev Pharmacol Toxicol. 2018;58:187-207. 10.1146/annurev-pharmtox-010716-105106 28992434 PMC5800772

[21] Hogg SJ , Beavis PA, Dawson MA, Johnstone RW. Targeting the epigenetic regulation of antitumour immunity. Nat Rev Drug Discov. 2020;19:776-800. 10.1038/s41573-020-0077-5 32929243

[22] Wu Z , Yang H, Duan S, Su Q, Cheng R, Hu T. Histone acetylation facilitates multidirectional pulp repair through neuregulin-1 mobilization. Stem Cells Transl Med. 2025;14:szaf022 10.1093/stcltm/szaf022 40580029 PMC12205360

[23] Lesot H , Smith AJ, Tziafas D, Begue-Kirn C, Cassidy N, Ruch JV. Biologically active molecules and dental tissue repair: a comparative review of reactionary and reparative dentinogenesis with the induction of odontoblast differentiation *in vitro*. Cells Mater. 1994;4:199-218.

[24] Neves VCM , Sharpe PT. Regulation of reactionary dentine formation. J Dent Res. 2018;97:416-422. 10.1177/0022034517743431 29185832

[25] Kitasako Y , Shibata S, Cox CF, Tagami J. Location, arrangement and possible function of interodontoblastic collagen fibres in association with calcium hydroxide-induced hard tissue bridges. Int Endod J. 2002;35:996-1004. 10.1046/j.1365-2591.2002.00606.x 12653318

[26] Jain A , Bahuguna R. Role of matrix metalloproteinases in dental caries, pulp and periapical inflammation: an overview. J Oral Biol Craniofac Res. 2015;5:212-218. 10.1016/j.jobcr.2015.06.015 26605147 PMC4623218

[27] Veis A. Mineral-matrix interactions in bone and dentin. J Bone Mine Res 1993;8:S493-S497. 10.1002/jbmr.5650081312 8122518

[28] Amir M , Jeevithan L, Barkat M, et al Advances in regenerative dentistry: A systematic review of harnessing wnt/β-catenin in dentin-pulp regeneration. Cells. 2024;13:1153. 10.3390/cells13131153 38995004 PMC11240772

[29] Zhao Y , Song L, Li M, et al Injectable CNPs/DMP1-loaded self-assembly hydrogel regulating inflammation of dental pulp stem cells for dentin regeneration. Mater Today Bio. 2024;24:100907. 10.1016/j.mtbio.2023.100907 PMC10758968 38170028

[30] Rajasekar V , Abdalla MM, Neelakantan P, Yiu CKY. Cellular dynamics and signalling mechanisms in dentine repair: a narrative review. Int Endod J. 2025;58:1354-1383. 10.1111/iej.14261 40491185

[31] Chen J , Xu H, Xia K, Cheng S, Zhang Q. Resolvin E1 accelerates pulp repair by regulating inflammation and stimulating dentin regeneration in dental pulp stem cells. Stem Cell Res Ther. 2021;12:75. 10.1186/s13287-021-02141-y 33482900 PMC7821538

[32] Sousa AB , Águas AP, Barbosa MA, Barbosa JN. Immunomodulatory biomaterial-based wound dressings advance the healing of chronic wounds via regulating macrophage behavior. Regen Biomater. 2022; 9: rbac065. 10.1093/rb/rbac065 36267154 PMC9566965

[33] Karkoutly M , Alnour A, Abu Hasna A, Nam OH, Al Kurdi S, Bshara N. Treatment outcomes of pulpotomy in primary molars utilizing 2.25% sodium hypochlorite gel: a randomized controlled trial. BMC Oral Health. 2025;25:1052. 10.1186/s12903-025-06438-9 40604659 PMC12224463

[34] Cunha D , Souza N, Moreira M, et al 3D-printed microgels supplemented with dentin matrix molecules as a novel biomaterial for direct pulp capping. Clin Oral Investig. 2023;27:1215-1225. 10.1007/s00784-022-04735-z PMC10171721 36287273

[35] Scheller EL , Chang J, Wang CY. Wnt/beta-catenin inhibits dental pulp stem cell differentiation. J Dent Res. 2008;87:126-130. 10.1177/154405910808700206 18218837 PMC2906770

[36] Gronthos S , Brahim J, Li W, et al Stem cell properties of human dental pulp stem cells. J Dent Res. 2002;81:531-535. 10.1177/154405910208100806 12147742

[37] Zannas AS , Jia M, Hafner K, et al Epigenetic upregulation of FKBP5 by aging and stress contributes to NF-κB-driven inflammation and cardiovascular risk. Proc Natl Acad Sci U.S.A. 2019;116:11370-11379. 10.1073/pnas.1816847116 31113877 PMC6561294

[38] Tian XY , Zhu B, Fang WC, et al FKBP5 regulates the osteogenesis of human adipose-derived mesenchymal stem cells. Curr Med Sci. 2024;44:1270-1279. 10.1007/s11596-024-2941-8 39586968

[39] Tang J , Li M, Chen Y, et al FKBP5 promotes osteogenic differentiation of mesenchymal stem cells through type-I interferon pathway inhibition. Cell Mol Life Sci. 2025;82:236. 10.1007/s00018-025-05754-1 40515831 PMC12167206

[40] Ruiz-Estevez M , Staats J, Paatela E, et al Promotion of myoblast differentiation by Fkbp5 via Cdk4 isomerization. Cell Rep. 2018;25:2537-2551.e8. 10.1016/j.celrep.2018.11.006 30485818 PMC6350781

[41] Gao S , Huang S, Zhang Y, et al The transcriptional regulator KLF15 is necessary for myoblast differentiation and muscle regeneration by activating FKBP5. J Biol Chem. 2023;299:105226. 10.1016/j.jbc.2023.105226 37673339 PMC10622842

[42] Chen Y , Jia M, Ge L, et al A negative feedback loop compromises NMD-mediated virus restriction by the autophagy pathway in plants. Adv Sci (Weinh.). 2024;11:e2400978. 10.1002/advs.202400978 39189522 PMC11348178

[43] Degtiar E , Fridman A, Gottlieb D, et al The feedback control of UPF3 is crucial for RNA surveillance in plants. Nucleic Acids Res. 2015;43:4219-4235. 10.1093/nar/gkv237 25820429 PMC4417159

[44] Zannas AS , Wiechmann T, Gassen NC, Binder EB. Gene-stress-epigenetic regulation of FKBP5: clinical and translational implications. Neuropsychopharmacology 2016;41:261-274. 10.1038/npp.2015.235 26250598 PMC4677131

[45] Fries G , Gassen N, Rein T. The FKBP51 glucocorticoid receptor co-chaperone: Regulation, function, and implications in health and disease. IJMS. 2017;18:2614. 10.3390/ijms18122614 29206196 PMC5751217

[46] Casagrande L , Demarco FF, Zhang Z, Araujo FB, Shi S, Nör JE. Dentin-derived BMP-2 and odontoblast differentiation. J Dent Res. 2010;89:603-608. 10.1177/0022034510364487 20351355

[47] Wang X , Lin C, Jin S, Wang Y, Peng Y, Wang X. Cannabidiol alleviates neuroinflammation and attenuates neuropathic pain via targeting FKBP5. Brain Behav Immun. 2023;111:365-375. 10.1016/j.bbi.2023.05.008 37196785

[48] Han K , Singh K, Rodman MJ, et al Fasting-induced FOXO4 blunts human CD4(+) T helper cell responsiveness. Nat Metab. 2021;3:318-326. 10.1038/s42255-021-00356-0 33723462 PMC7990708

[49] Chen X , Song QL, Wang JY, et al FKBP5 regulates trophoblast-macrophage crosstalk in recurrent spontaneous abortion through PI3K/AKT and NF-κB signaling pathways. Free Radic Biol Med. 2023;209:55-69. 10.1016/j.freeradbiomed.2023.10.380 37827456

[50] Kremer TL , Chen J, Buhl A, et al Multimodal associations of FKBP5 methylation with emotion-regulatory brain circuits. Biol Psychiatry. 2024;96:858-867. 10.1016/j.biopsych.2024.03.003 38460581

[51] Klinger-König J , Hertel J, Van der Auwera S, et al Methylation of the FKBP5 gene in association with FKBP5 genotypes, childhood maltreatment, and depression. Neuropsychopharmacology 2019;44:930-938. 10.1038/s41386-019-0319-6 30700816 PMC6461917

[52] Mihaljevic M , Franic D, Soldatovic I, et al The FKBP5 genotype and childhood trauma effects on FKBP5 DNA methylation in patients with psychosis, their unaffected siblings, and healthy controls. Psychoneuroendocrinology. 2021;128:105205. 10.1016/j.psyneuen.2021.105205 33933892

[53] Girgenti MJ , Wang J, Ji D, et al Traumatic Stress Brain Research Group. Transcriptomic organization of the human brain in post-traumatic stress disorder. Nat Neurosci. 2021;24:24-33. 10.1038/s41593-020-00748-7 33349712

[54] Klengel T , Mehta D, Anacker C, et al Allele-specific FKBP5 DNA demethylation mediates gene-childhood trauma interactions. Nat Neurosci. 2013;16:33-41. 10.1038/nn.3275 23201972 PMC4136922

