# Supplementary material for: Histone acetylation promotes FKBP5 to increase reparative dentin formation in pulp repair
Source: Stem Cells Transl Med. 2026 Jul 28;15(8):szag047. doi: 10.1093/stcltm/szag047 (PMC13415464; doi:10.1093/stcltm/szag047)
Supplement: szag047_Supplementary_Data [file szag047_supplementary_data.docx]

**Title:** **Histone acetylation promotes FKBP5 to increase reparative dentin formation in pulp repair**

**Authors:** Shaoying Duan^1,†^，Qianqian Su ^1,†^，Hui Yang^1,†^，Xinlei Hu^2^，Aopeng Zhang^1^，Tao Hu^1,*^，Dan Xie^2,*^，Ran Cheng^1,*^

†These authors contributed equally to this work.

*Corresponding authors:

Email: hutao@scu.edu.cn (T, Hu), [danxie@scu.edu.cn(D,Xie)](mailto:danxie@scu.edu.cn(D,Xie)), chengran@scu.edu.cn (R,Cheng)

Address: Tao Hu, State Key Laboratory of Oral Diseases & National Center for Stomatology & National Clinical Research Center for Oral Diseases & Frontier Innovation Center for Dental Medicine Plus, West China Hospital of Stomatology, Sichuan University, Chengdu, 610041, China.

**Affiliations:**

^1^State Key Laboratory of Oral Diseases & National Center for Stomatology & National Clinical Research Center for Oral Diseases & Frontier Innovation Center for Dental Medicine Plus, West China Hospital of Stomatology, Sichuan University; Chengdu, China, 610041

^2^Frontier Science Center for Disease Molecular Network, State Key Laboratory of Biotherapy, West China Hospital, Sichuan University; Chengdu, China, 610041

**Author contributions:**

Shaoying Duan: Investigation, Formal analysis, Visualization, Writing – original draft, Writing –review & editing. Qianqian Su: Investigation, Formal analysis, Visualization, Writing – original draft, Writing –review & editing. Hui Yang: Conceptualization, Funding acquisition, Project administration, Supervision, Writing – review & editing.Xinlei Hu: Formal analysis, Visualization. Aopeng Zhang: Visualization. Tao Hu: Conceptualization, Data curation, Funding acquisition, Supervision, Writing –review & editing. Dan Xie: Conceptualization, Supervision, Writing – review & editing. Ran Cheng: Conceptualization, Data curation, Funding acquisition, Methodology, Supervision, Writing – original draft, Writing –review & editing.

**Acknowledgments:**

This study was supported in part by the National Natural Science Foundation of China (grant ID: U20A20365 and 81970948), and Sichuan Science and Technology Program (grant ID: 2024NSFSC0543 and 2024YFHZ0043). The schematic diagrams in figures were created with BioRender.com.

**Keywords**

H3K9ac, H3K27ac, FKBP5, SAHA, dental pulp stem cells

**Supplemental material**

1. **Supplemental materials and methods**

**Culture of human dental pulp stem cells (DPSCs)**

Human premolars or third molars (ages 12–18) were collected with informed consent and ethics approval. Dental pulp was isolated under sterile conditions, digested with type I collagenase, and cultured in DMEM with 20% FBS and 1% antibiotics at 37°C with 5% CO₂ to obtain DPSCs. In this study, DPSCs were seeded in 6-well plates at a density of 1×10^6^ cells/well. Cells were stimulated with LPS at a concentration of 1 μg/mL to establish an *in vitro* dental pulp injury model. OD induction medium (DMEM medium, 10 nmol/L sodium β-glycerophosphate, 50 μg/mL ascorbic acid, 10 mmol/L dexamethasone) was added for OD culture to establish an *in vitro* mineralization model. Based on the *in vitro* dental pulp injury and OD models, 1 μM SAHA (S1047, Selleck, USA) and 10 μM C646 (S7152, Selleck) were used for histone acetylation intervention, respectively.

**Flow Cytometry Analysis**

Third-passage hDPSCs were harvested, washed twice with PBS, and resuspended in 100 μL PBS. Cells were incubated with fluorochrome-conjugated antibodies against CD29, CD34, CD44, CD90, and CD105 for 30 minutes at 4 °C in the dark. After incubation, cells were washed twice with PBS, centrifuged at 1500 rpm for 5 minutes, and finally resuspended in 500 μL PBS. Surface marker expression was analyzed using a Beckman CytoFLEX flow cytometer (Beckman Coulter, USA).

**Real-Time Quantitative Polymerase Chain Reaction (qPCR)**

hDPSCs were seeded at a density of 1×10⁶ cells per well in 6-well plates and treated with LPS, or induced for odontogenic differentiation. Total RNA was extracted using the Animal RNA Extraction Kit (R0026, Beyotime, China), and reverse-transcribed into cDNA using the Reverse Transcription Kit (11141ES60, Yeason, China). qPCR was performed using TB Green® Premix Ex Taq™ II FAST (CN830S, Takara, Japan) on a real-time PCR system. β-actin was used as the internal reference gene. Relative gene expression was calculated using the 2^^−ΔΔCt^ method. Primer sequences are detailed in Supplementary Table S1.

**Table S1 (Primers)**

| **Gene name** | **Forward primer (5’-3’)** | **Reverse primer (5’-3’)** |
| --- | --- | --- |
| *FKBP5* | GAATATCCCTCTCCTTTCCGTTTGG | ATATGGCTCGGCTGGCAGTC |
| *RUNX2* | CATGTCCCTCGGTATGTCCG | ACTCTGGCTTTGGGAAGAGC |
| *OSX* | CCTCTGCGGGACTCAACAAC | AGCCCATTAGTGCTTGTAAAGG |
| *ALP* | CAACAGGGTAGATTTCTCTTGG | GGTCAGATCCAGAATGTTCC |
| *IL-1β* | TTATTACAGTGGCAATGAGGATGAC | CCTGAAGCCCTTGCTGTAGTG |
| *IL-6* | ACTCACCTCTTCAGAACGAATTG | CCATCTTTGGAAGGTTCAGGTTG |
| *IL-8* | ACTCCTTGGCAAAACTGCAC | AAGAAACCACCGGAAGGAAC |
| *β-ACTIN* | CCACGAAACTACCTTCAACTCC | GTGATCTCCTTCTGCATCCTGT |

**Clinical Sample Collection**

Normal, carious, and pulpitis-affected teeth were collected from patients at the West China Hospital of Stomatology, Sichuan University, with informed consent obtained from all participants or their legal guardians. Inclusion criteria were as follows: the caries group exhibited visible dental caries on clinical examination, no overt pulp symptoms, and radiographs indicating low-density lesions confined to superficial dentin; the pulpitis group presented with clinical symptoms of pulpitis and radiographic evidence of lesion extension into the pulp; normal teeth (premolars or third molars) were extracted for orthodontic purposes. Exclusion criteria included systemic disease or use of antibiotics or nonsteroidal anti-inflammatory drugs within the preceding three months. All samples were rinsed with saline and immediately fixed in 4% paraformaldehyde.

**Micro-CT Analysis**

Maxillary samples from the mineralization model at days 7 and 28 were scanned using a Scanco μCT45 system (Scanco Medical, Switzerland) with a resolution of 10 μm, 55 kVp voltage, 145 μA current, and 250 ms exposure time. The region of interest was the crown of the maxillary first molar. Dentin formation within the pulp cavity was quantified by measuring bone mineral density (BMD), bone volume fraction (BV/TV), trabecular number (Tb.N), trabecular thickness (Tb.Th), and trabecular separation (Tb.Sp). Increased BMD, BV/TV, Tb.N, and Tb.Th, along with decreased Tb.Sp, indicated enhanced dentin deposition. Representative μCT cross-sectional images were used to visualize the extent of mineralization.

**Hematoxylin and Eosin (HE) Staining**

Tissue sections were processed using a commercial H&E staining kit (G1120, Solarbio, China). After deparaffinization and rehydration, nuclei were stained with hematoxylin and cytoplasm with eosin. Sections were then dehydrated, cleared, mounted, and imaged under a light microscope (Leica DM2000, Leica Microsystems, Germany).

**Immunofluorescence (IF) Staining**

For conventional immunofluorescence, antigen retrieval was performed using Tris-EDTA buffer, followed by blocking with 5% BSA for 30 minutes at 37°C. Tissue sections were incubated overnight at 4℃ with primary antibodies (e.g., FKBP5, DMP1, DSPP), then incubated with appropriate fluorescent-conjugated secondary antibodies for 30 minutes at 37℃. Nuclei were counterstained with DAPI for 10 minutes at room temperature. Fluorescence images were acquired using a fluorescence microscope (Olympus BX53, Olympus Corporation, Japan).

**Multiplex Immunohistochemistry (mIHC)**

Multiplex immunohistochemical staining was performed using the Multiplex Fluorescence IHC Staining Kit (abs50012, Absin, China) according to the manufacturer's instructions. After blocking with 5% BSA for 30 minutes at room temperature, sections were incubated with the first primary antibody overnight at 4℃, followed by HRP-conjugated secondary antibody incubation for 10 minutes at room temperature. Tyramide signal amplification working solution was then applied for 10 minutes at room temperature. Antibody stripping was conducted after each round, and subsequent antibody cycles were repeated using the same procedure. DAPI was used for nuclear counterstaining for 10 minutes at room temperature. Three-color mIHC panels included FKBP5, H3K9ac, and H3K27ac; FKBP5, DSPP, and DMP1; and FKBP5, IL-1β, and IL-6.

**Immunohistochemistry (IHC) Staining**

IHC was performed using a commercial staining kit (E-IR-R217, Elabscience Biotechnology Co., LTD, China) according to the manufacturer's protocol. After blocking with goat serum for 30 minutes at 37℃, tissue sections were incubated overnight at 4℃ with primary antibodies (FKBP5, H3K9ac, H3K27ac). The following day, sections were incubated with HRP-conjugated anti-rabbit or anti-mouse secondary antibodies for 30 minutes at 37℃. Images were acquired using a light microscope (Leica DM2000, Leica Microsystems, Germany), and relative expression levels were quantified as integrated optical density (IOD)/area using Image-Pro Plus 6.0 software (Media Cybernetics, Inc., USA).

**Real-Time Quantitative Polymerase Chain Reaction (qPCR)**

hDPSCs were seeded at a density of 1×10⁶ cells per well in 6-well plates and treated with LPS, or induced for odontogenic differentiation. Total RNA was extracted using the Animal RNA Extraction Kit (R0026, Beyotime, China), and reverse-transcribed into cDNA using the Reverse Transcription Kit (11141ES60, Yeason, China). qPCR was performed using TB Green® Premix Ex Taq™ II FAST (CN830S, Takara, Japan) on a real-time PCR system. β-actin was used as the internal reference gene. Relative gene expression was calculated using the 2^^−ΔΔCt^ method. Primer sequences are detailed in Supplementary Table S1.

**Western Blotting**

DPSCs were seeded at a density of 1×10⁶ cells per well in 6-well plates and treated with LPS, odontogenic differentiation medium, SAHA, or C646. Total protein was extracted using the Total Protein Extraction Kit (PE001, Signalway Antibody, USA). Equal amounts of protein were separated by SDS-PAGE (ET15420gel, ACE Biotechnology, China) and transferred onto polyvinylidene difluoride (PVDF) membranes (GE Healthcare Life Science, USA). Membranes were blocked with 5% BSA for 1 hour at room temperature and incubated overnight at 4℃ with primary antibodies against FKBP5, H3K9ac, H3K27ac, IL-1β, IL-6, IL-8, DSPP, DMP1, and β-Tubulin. The following day, membranes were incubated with appropriate secondary antibodies for 1 hour at room temperature. Signal detection was performed using chemiluminescent substrate, and images were acquired using a ChemiDoc imaging system (Bio-Rad, USA).

**Alkaline phosphatase (ALP) staining and activity assay**

DPSCs were seeded at a density of 1×10⁶ cells per well in 6-well plates and subjected to OD induction for 7 days. ALP staining was performed using the BCIP/NBT Alkaline Phosphatase Color Development Kit (C3206, Beyotime, China) according to the manufacturer’s instructions. After incubation with the BCIP/NBT working solution, the reaction was terminated after 5 minutes at room temperature. Stained cells were observed under a microscope (Olympus 1X7, Japan).

**Alizarin red S staining and measurement of calcium concentration**

DPSCs were seeded at a density of 1×10⁶ cells per well in 6-well plates and subjected to OD induction. After induction, cells were incubated with Alizarin Red S staining solution (G1450, Solarbio, China) for 30 minutes at room temperature to assess calcium deposition. Stained mineralized nodules were visualized under a microscope (Olympus 1X7, Japan). For quantification, the bound dye was eluted using 10% cetylpyridinium chloride in double-distilled water, and the absorbance was measured at 570 nm using a microplate reader (SpectraMax iD3, Molecular Devices, USA).

**Preparation of human-treated dentin matrix (TDM)**

Human premolars extracted for orthodontic purposes or intact third molars were selected. Roots were sectioned into 3-5 mm fragments, and the outer dentin layer was lightly abraded with a dental handpiece. Samples were ultrasonically cleaned and decalcified sequentially with 17%, 10%, and 5% EDTA, each for 20 minutes. They were then disinfected in iodophor for 30 minutes, rinsed three times with PBS for 5 minutes each, and finally treated with 5.25% sodium hypochlorite for 10 to 15 minutes.

**Cell Viability Assay**

Cell viability was assessed using the CCK-8 kit (K1018, APE×BIO, USA) according to the manufacturer’s instructions. DPSCs were seeded in 96-well plates at a density of 5×10³ cells per well in 100 μL of complete medium and incubated at 37°C with 5% CO₂ for 24 hours. Subsequently, cells were treated with SAHA (S1047, Selleck, USA) at 0.5 μM, 1 μM, 5 μM, or 10 μM, or with C646 (S7152, Selleck, USA) at 1 μM, 5 μM, 10 μM, or 20 μM. Cell viability was evaluated after 24, 48, and 72 hours of treatment.At each time point, 10% CCK-8 reagent was added to each well and incubated for an additional 2 hours. Absorbance at 450 nm was measured using a microplate reader (SpectraMax iD3, Molecular Devices, USA). Wells containing only medium and CCK-8 reagent were used as blank controls. Cell proliferation rate (%) was calculated as: (ODₓ-OD_blank)/(OD_control-OD_blank)×100.

**Chromatin Immunoprecipitation Sequencing (ChIP-seq) Library preparation and Sequencing**

hDPSCs were harvested at 7 days after odontogenic induction. ChIP DNA degradation and contamination was monitored on agarose gels. Protein-DNA complexes in immobilized living cells are randomly cleaved into chromatin fragments, and immunological methods are applied to precipitate the fragments, enrich for the target protein-bound DNA fragments, and purify and assay them. DNA purity was checked using the NanoPhotometer® spectrophotometer (IMPLEN, CA, USA). DNA concentration was measured using Qubit® DNA Assay Kit in Qubit® 3.0 Flurometer (Life Technologies, CA, USA). The purified DNA was used for ChIP-seq library preparation. The library was constructed by Novogene Corporation (Beijing, China). Subsequently, pair-end sequencing of sample was performed on Illumina platform (Illumina, CA, USA). Library quality was assessed on the Agilent Bioanalyzer 2100 system.Sequencing libraries were pooled based on effective concentration and target sequencing depth. Bioinformatic analyses included quality filtering, alignment to the reference genome, and peak calling to identify genomic regions enriched for H3K9ac and H3K27ac.

**Table S2 (Antibodies)**

| Antibodies | Catalog  Number | Supplier | Application |
| --- | --- | --- | --- |
| rabbit anti-FKBP5 | ab126715 | Abcam, England | IHC, IF, mIHC, WB |
| rabbit anti-H3K9ac | YK0006 | Immonoway, USA | IHC, mIHC |
| mouse anti-H3K27ac | HA500046 | Huabio, China | IHC, mIHC |
| rabbit anti-IL-1β | TA 5103 | Abmart, China | IF, mIHC |
| rabbit anti-IL-6 | R1412-2 | Huabio, China | mIHC |
| rabbit anti-IL-8 | R1511-15 | Huabio, China | IF |
| mouse anti-DSPP | sc-73632 | Santa Cruz, USA | IF, mIHC |
| mouse anti-DMP1 | sc73633 | Santa Cruz, USA | IF, mIHC |
| rabbit anti-H3K9ac | YK0006 | Immonoway, USA | WB |
| mouse anti-H3K27ac | HA500046 | Huabio, China | WB |
| rabbit anti-IL-1β | TA5103 | Abmart, China | WB |
| rabbit anti-IL-8 | R1511-15 | Huabio, China | WB |
| mouse anti-DSPP | sc-73632 | Santa Cruz, USA | WB |
| mouse anti-DMP1 | sc73633 | Santa Cruz, USA | WB |
| mouse anti-β-tubulin | 44032 | Signalway Antibody, USA | WB |
| rabbit anti-conecexin43 | 26980-1-AP | Proteintech, USA | WB |
| goat anti-mouse IgG | L3032 | Signalway Antibody, USA | WB |
| goat anti-rabbit IgG | (L3012 | Signalway Antibody, USA | WB |
| rabbit anti-ZO-1 | YN1410 | Immonoway, USA | mIHC |
| rabbit anti-H3K9ac  ChIP grade | ab32129 | Abcam, England | ChIP-qPCR |
| rabbit anti-H3K27ac  ChIP grade | ab4729 | Abcam, England | ChIP-qPCR |
| mouse anti-CD29-APC | 17-0299-41 | Thermo Fisher, USA | Flow cytometry |
| mouse anti-CD34-FITC | 343603 | Biolegend, USA | Flow cytometry |
| mouse anti-CD44-FITC | 338803 | Biolegend, USA | Flow cytometry |
| mouse anti-CD45-PE | 368509 | Biolegend, USA | Flow cytometry |
| mouse anti-CD90-APC | 17-0909-41 | Thermo Fisher, USA | Flow cytometry |
| mouse anti-CD105-APC | 17-1057-41 | Thermo Fisher, USA | Flow cytometry |
| 488 goat anti-mouse IgG | A11011 | Invitrogen, Carlsbad, USA | IF |
| 488 goat anti-rabbit IgG | A11008 | Invitrogen, Carlsbad, USA | IF |

**Supplemental figures**


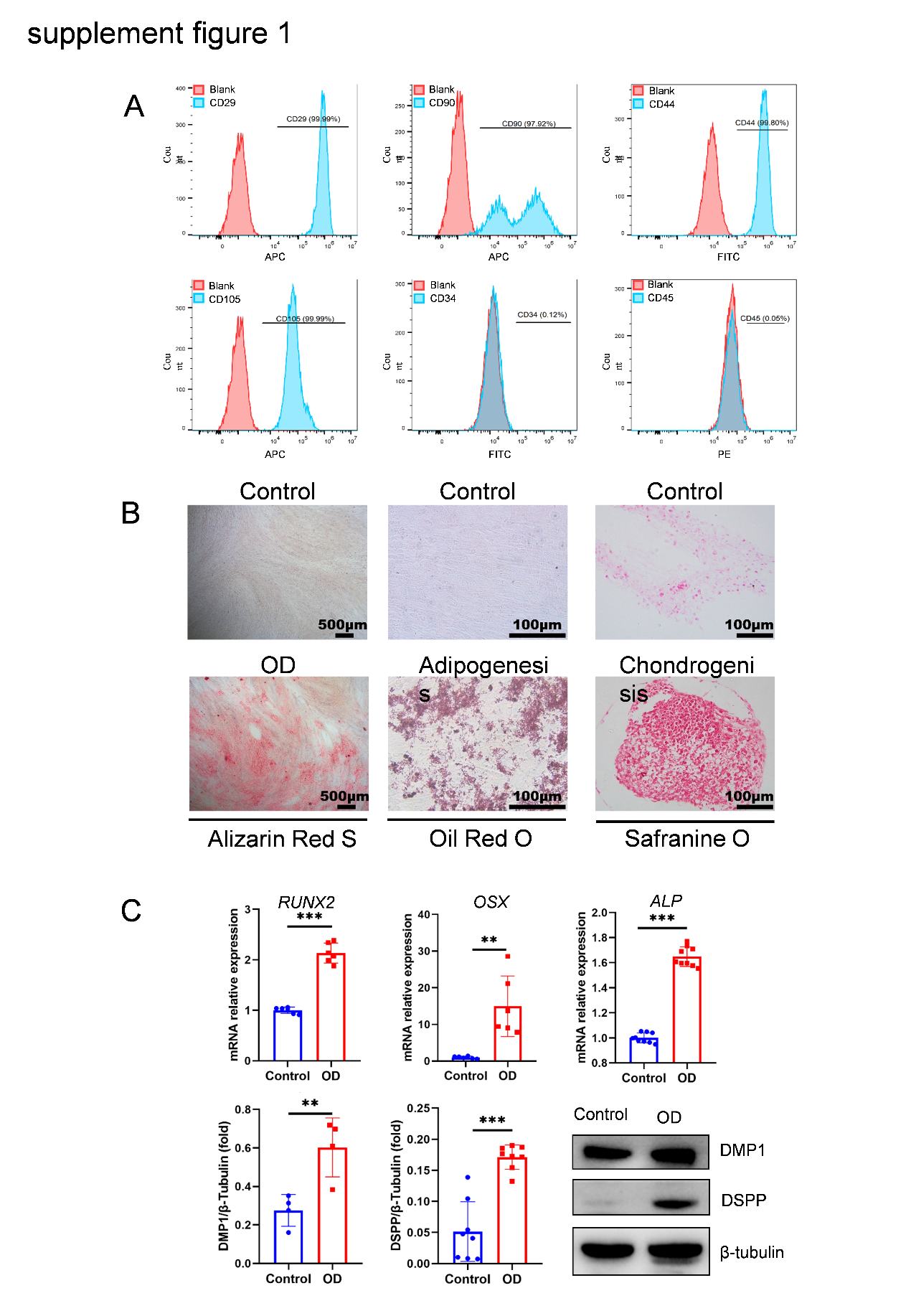


Figure S1. A. DPSCs were cultured by collagenase digestion method, the cells showed fibroblast-like morphology, scale bar: 500 μm. B. Detection of markers of DPSCs by flow cytometry, the results showed that the cells originated from mesenchymal layer. C. Alizarin red S staining after OD induction, oil red O staining after lipogenic induction, and fannin red O staining after cartilage inducion, scale bar: 100 μm. (D) Alizarin Red S staining after OD induced, scale bar: 100μm. (E) RUNX2, OSX, and ALP mRNA expression of DPSCs after OD induction for 3 days (*N*=6-9; **, *P*<0.01; ****P*<0.001). (F) DMP1 and DSPP protein expressions of DPSCs after OD induction for 14 days ( *N*=4-8; **, *P*<0.01; ****P*<0.001)


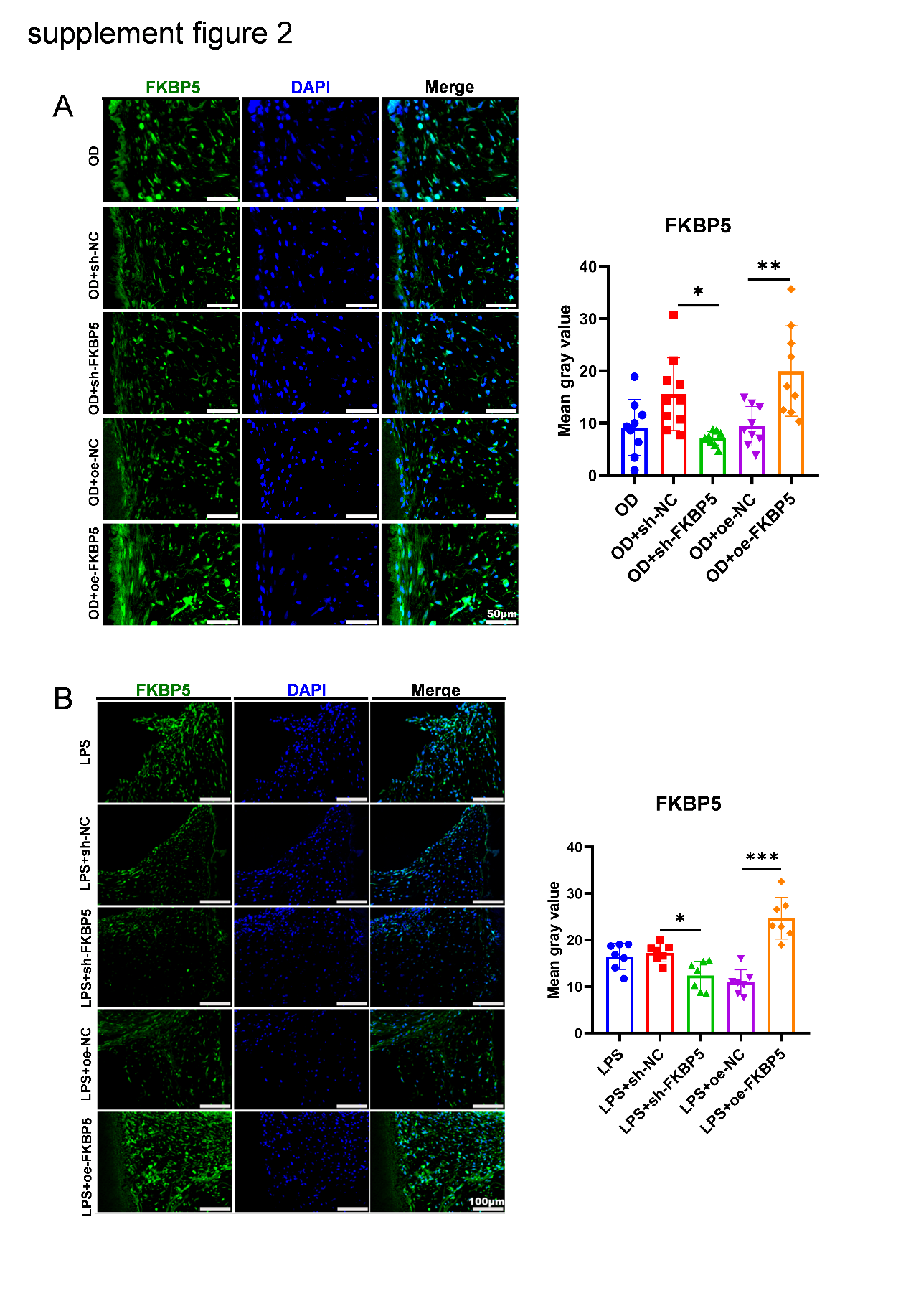


Figure S2. The *in vivo* ectopic model of TDM established by FKBP5 knockdown/overexpressing in the dorsal subcutis of nude mice. A. The expression of FKBP5 after OD induction. Scale bar: 50 μm (*N*=9; * *P*< 0.05). B. the Expression of FKBP5 after LPS stimulation. scale bar: 100 μm (*N*=7; * *P*< 0.05; **** *P* < 0.001).


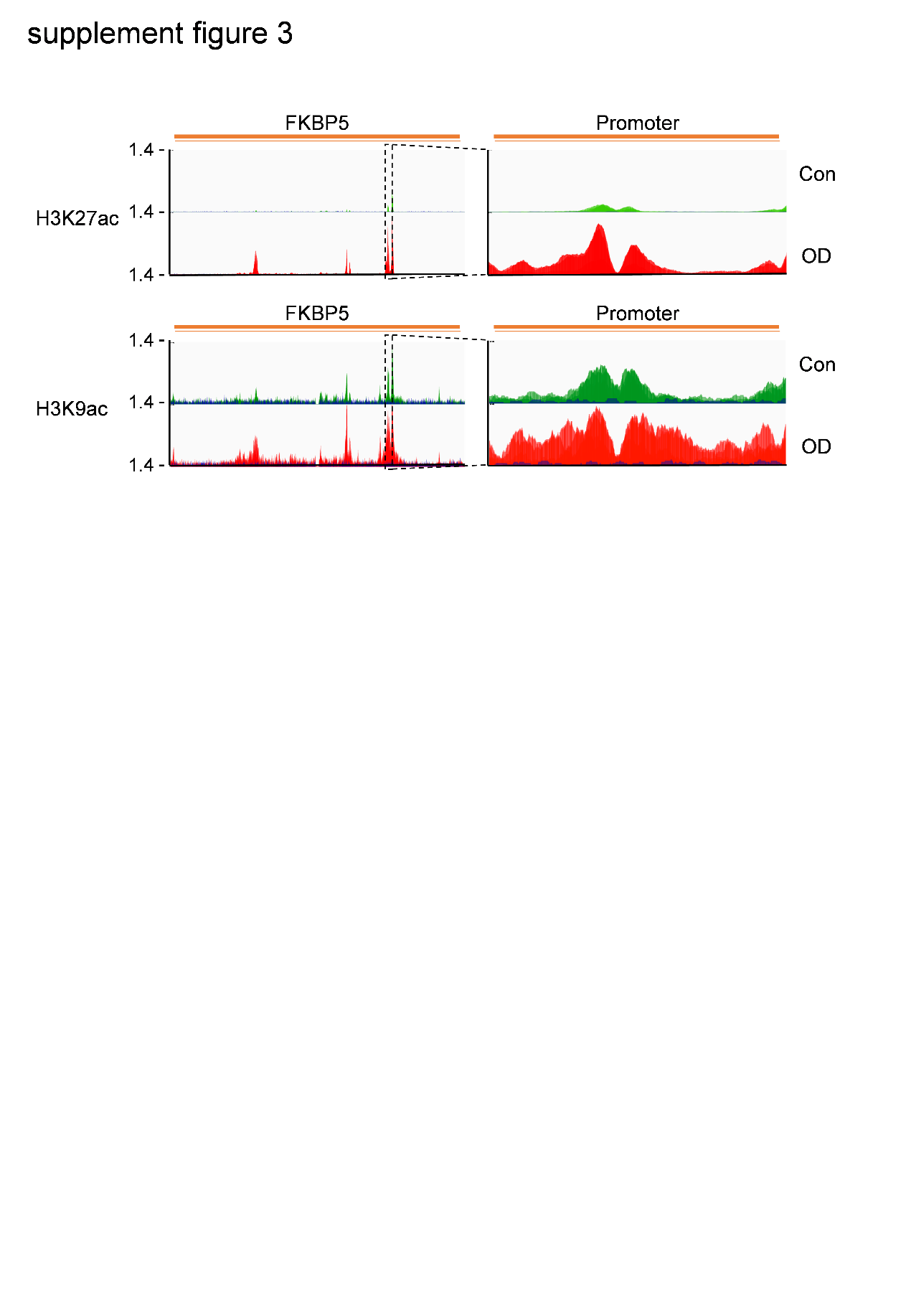


Figure S3. A.Lentiviral vectors for dCas9-P300 and sgRNA. B. Design and synthesis of two distinct sgRNA sequences targeting the peak signals in the variably accessible region of the FKBP5 promoter.


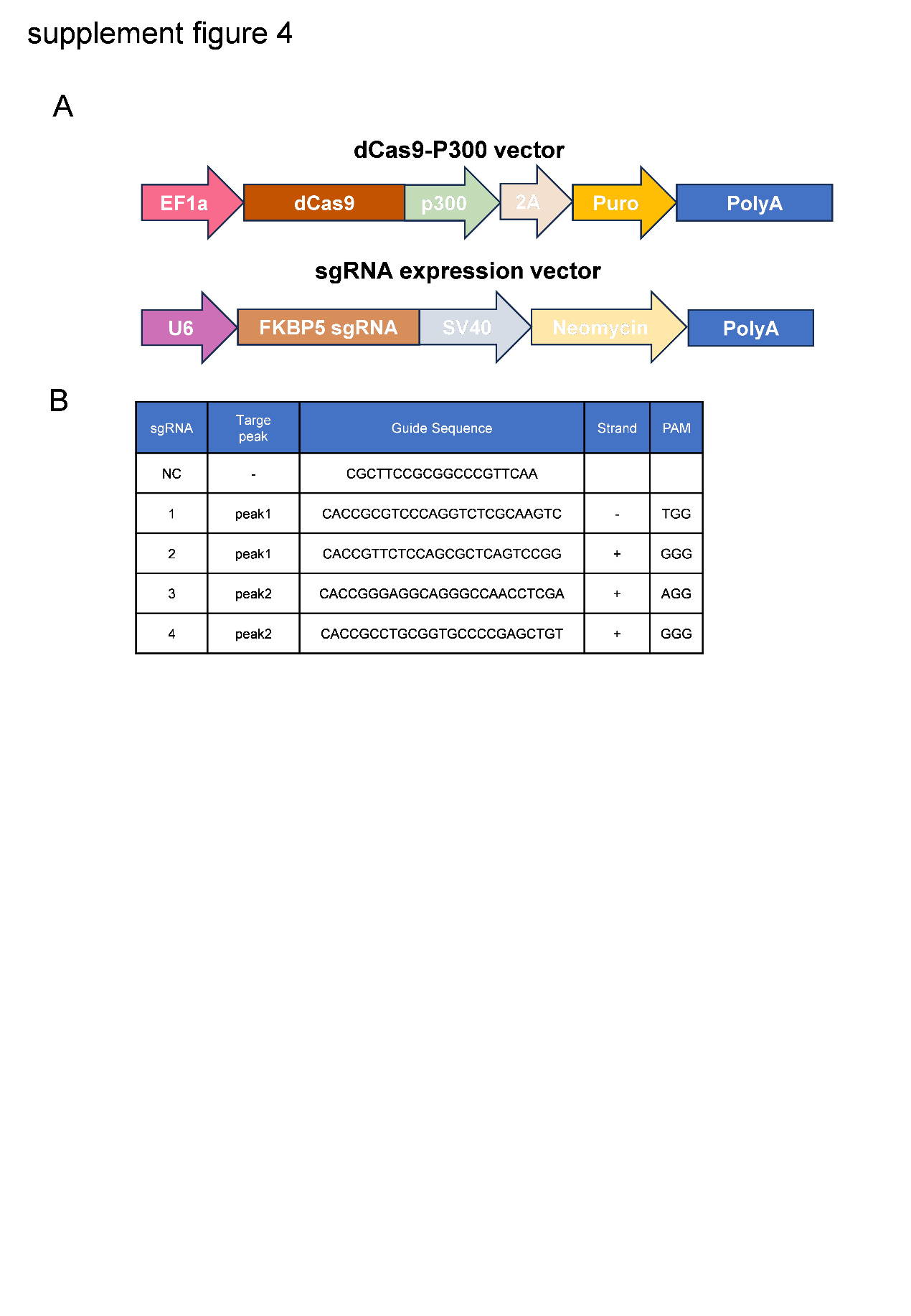


Figure S4. CHIP-seq reveals a histone acetylation alteration of the FKBP5 gene and its promoter region at H3K27 and H3K9 locations in both control and OD groups.


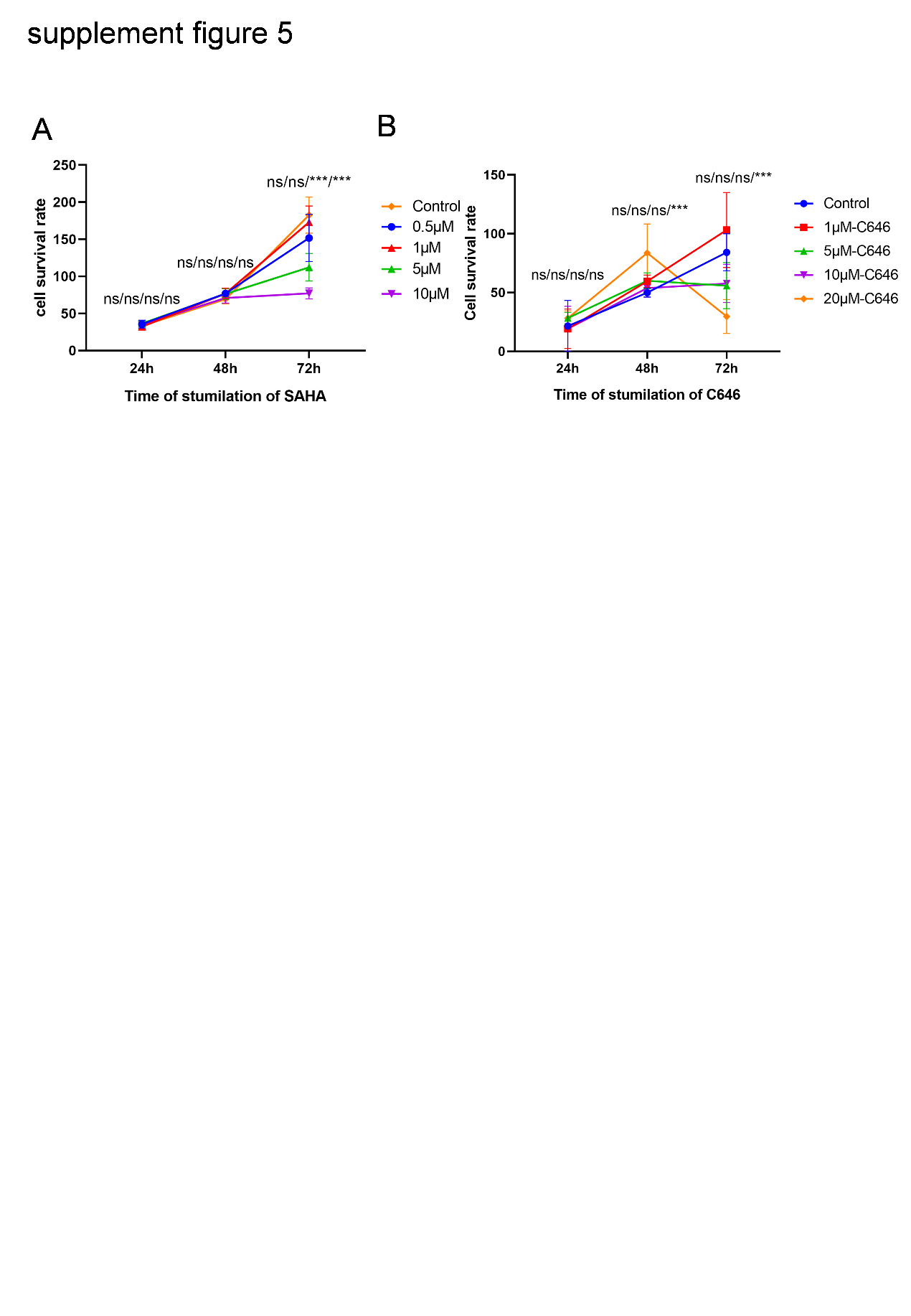


Figure S5. A. The CCK-8 assay of different concentrations of SAHA in DPSCs. B. The CCK-8 assay of different concentrations of C646 in DPSCs.


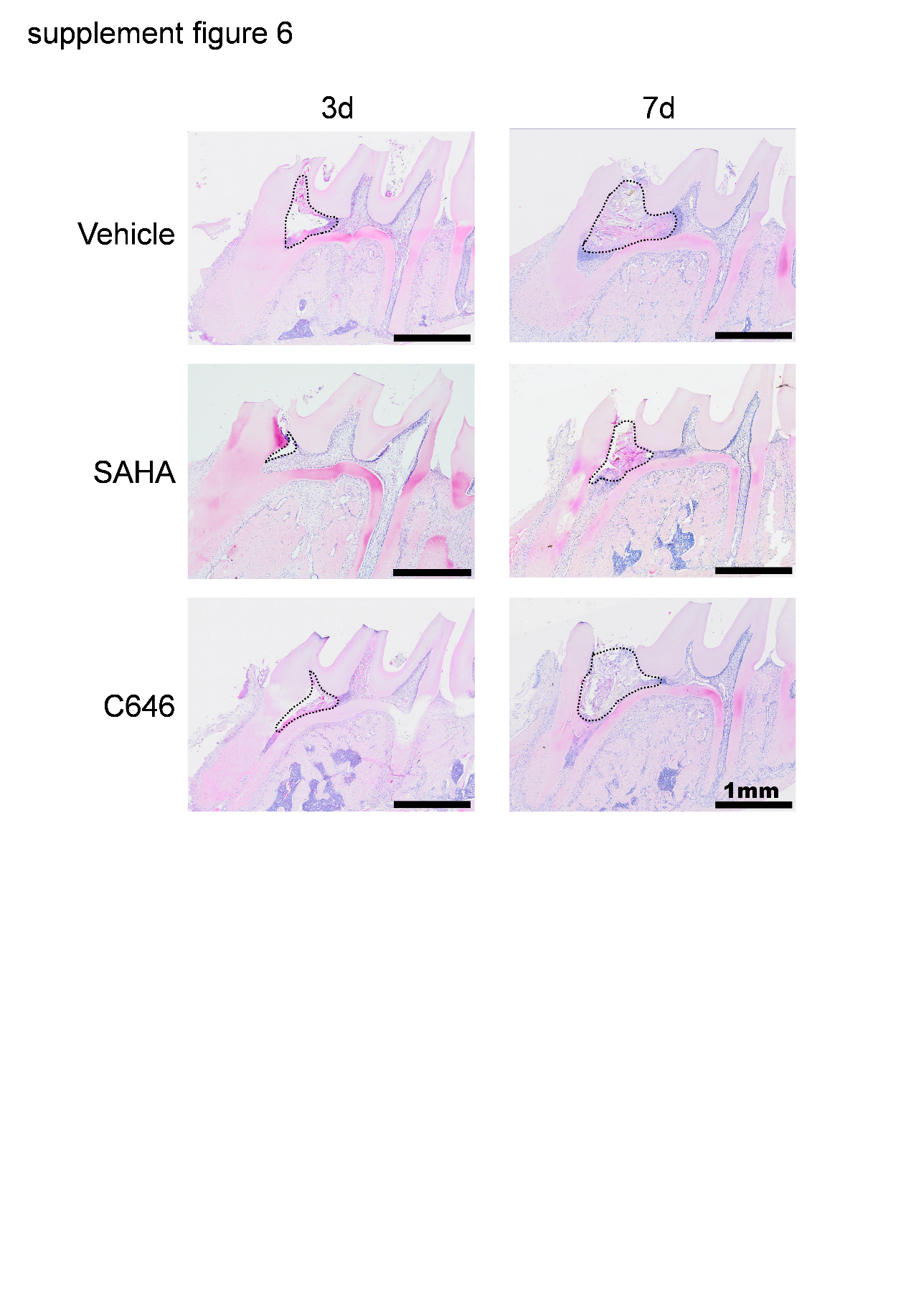


Figure S6. HE staining of the necrotic regions of pulpitis in rats after histone acetylation intervention . Black dashed box: region of pulp necrosis, scale bar: 1 mm


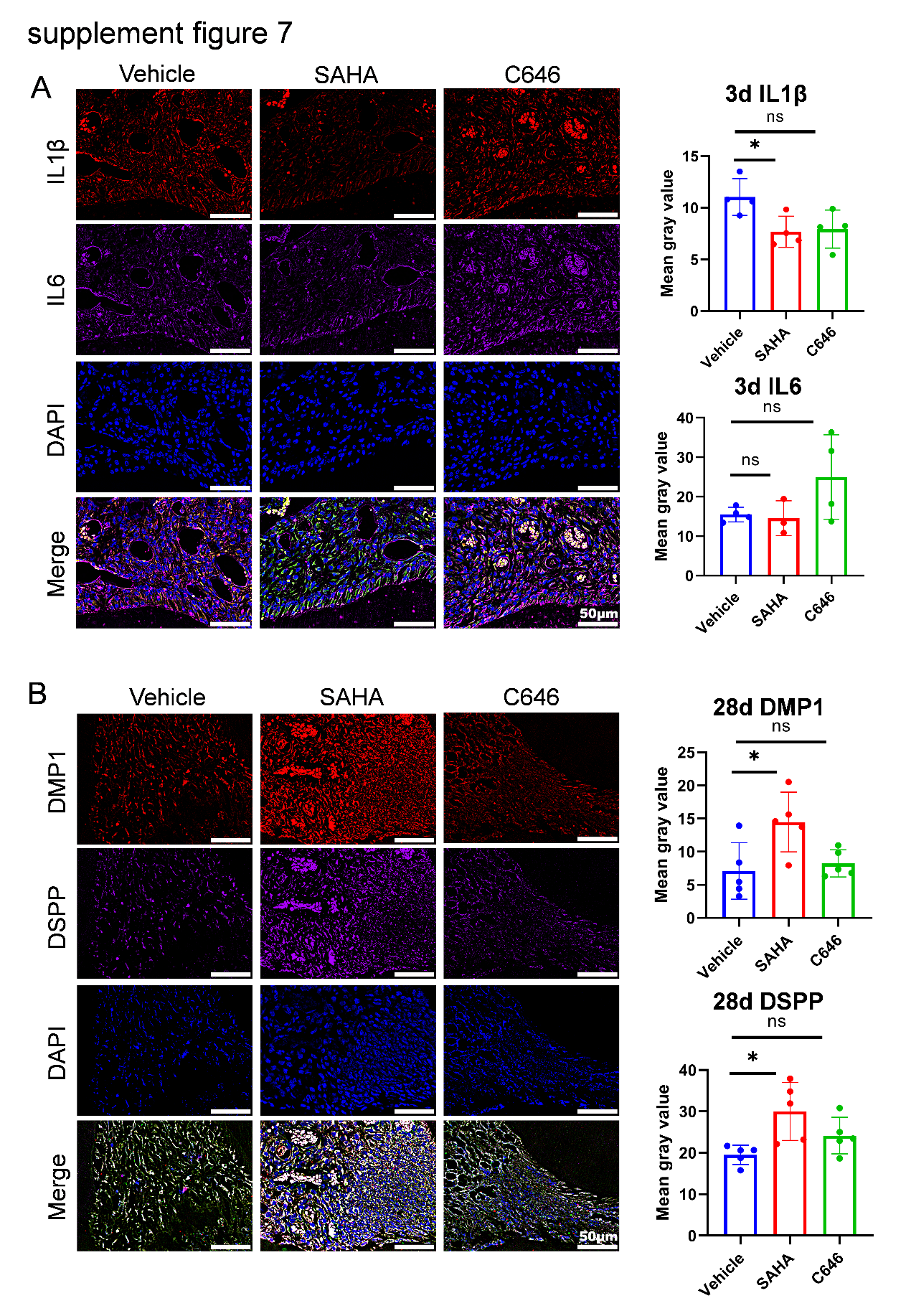


Figure S7. A. Multiple immunofluorescent staining of IL1β and IL6 in rat pulp injury model of 28 days. (*N*=3-4; ns, not significant; * *P*< 0.05). Scale bar: 50 μm. B. Multiple immunofluorescent staining of DMP1 and DSPP in rat pulp injury model of 28 days (*N*=3-4; ns, not significant; * *P*< 0.05). Scale bar: 50 μm.
